# Thromboinflammatory challenges in stroke pathophysiology

**DOI:** 10.1007/s00281-023-00994-4

**Published:** 2023-06-05

**Authors:** R.D Szepanowski, S Haupeltshofer, S.E Vonhof, B Frank, C Kleinschnitz, A.I Casas

**Affiliations:** 1 Department of Neurology, University Hospital Essen, Essen, Germany; 2 Center for Translational Neuro- and Behavioral Sciences (C-TNBS), Essen, Germany; 3 Department of Pharmacology and Personalised Medicine, Faculty of Health, Medicine, and Life Sciences, Maastricht University, Maastricht, The Netherlands

**Keywords:** Brain ischemia, Thromboinflammation, Stroke recovery, Platelet, Neuroinflammation

## Abstract

Despite years of encouraging translational research, ischemic stroke still remains as one of the highest unmet medical needs nowadays, causing a tremendous burden to health care systems worldwide. Following an ischemic insult, a complex signaling pathway emerges leading to highly interconnected thrombotic as well as neuroinflammatory signatures, the so-called thromboinflammatory cascade. Here, we thoroughly review the cell-specific and time-dependent role of different immune cell types, i.e., neutrophils, macrophages, T and B cells, as key thromboinflammatory mediators modulating the neuroinflammatory response upon stroke. Similarly, the relevance of platelets and their tight crosstalk with a variety of immune cells highlights the relevance of this cell-cell interaction during microvascular dysfunction, neovascularization, and cellular adhesion. Ultimately, we provide an up-to-date overview of therapeutic approaches mechanistically targeting thromboinflammation currently under clinical translation, especially focusing on phase I to III clinical trials.

## Introduction

Despite continuous improvements in stroke care over the past decades, contraindications of the currently approved recanalization therapies, such as an increased bleeding risk or the occlusion of only small vessels, prevent ~70% of all stroke patients to receive either intravenous thrombolysis with recombinant tissue plasminogen activator (rt-PA, Alteplase) or endovascular therapy (EVT), i.e., mechanical thrombectomy [1]. Moreover, ~30% of patients receiving intravenous thrombolysis experience re-occlusion events, therefore early secondary prophylaxis treatment is also a major therapeutic strategy in acute stroke treatment [2]. This risk of recurrent strokes after an initial event is also increased by other risk factors such as hypertension, diabetes, hyperlipidemia, and atrial fibrillation [3].

Even if the clinical outcome for the individual patient is still improved compared to patients receiving no recanalization therapies, thrombolysis-related complications display a major issue. The most severe complication as well as a contraindication for intravenous thrombolysis is the increased risk for intracerebral hemorrhage, especially if recanalization is performed too late. Even though this complication is rare and affects only ~5% of patients after rt-PA treatment, unfortunately leading to severe clinical scenarios including longer periods of hospitalization, and a 5-fold increase risk of disability or palliative care at discharge from the hospital [4, 5]. Similarly, ~50% of thrombectomized patients still present suboptimal outcomes after a large vessel occlusion despite this highly effective therapeutic strategy [6]. In patients neither eligible for pharmacological nor mechanical procedures, spontaneous recanalization often occurs. However, in case the vessel occlusion persists over time, the risk of unforeseeable infarct growth and therefore worsened clinical outcome significantly increases. As a result, patients recovering from an ischemic event often suffer from any kind of post-stroke disability. The fraction of these patients is highly variable, ranging from 24 to 96%, depending on the socio-economical composition of the cohort, the time of follow-up measurement as well as the scales used for evaluation. Overall, ~30% of patients experience various types of disability, including physical impairment, cognitive dysfunction, loss of overall quality of life, memory loss, depression or anxiety [7–10]. This severe impairment in daily quality of life entails a major burden in health care costs reaching ~26€ billion in Europe per year not including social care, productivity loss, and private caretaking, altogether close to ~60€ billion yearly [11]. Therefore, novel cerebroprotective approaches improving post-stroke outcomes are yet in urgent need.

Upon brain ischemia, a complex pathophysiological cascade is initiated involving both thrombotic and inflammatory pathways acting as key contributors to ischemic damage. Indeed, following the ischemic event, microvascular thrombosis resulting in poor cerebral blood flow directly causes neurological deficits and worsened long-term prognosis [12]. At the same time, a strong inflammatory response occurs causing cytokine overproduction, leukocyte infiltration, and generalized tissue damage surrounding the infarcted area. Therefore, the relevance of the complex interplay between thrombotic and inflammatory events highlights thromboinflammation as a key contributor to post-stroke damage [13].

Platelets, frequently considered the main contributor to ischemic stroke, directly modulate thrombosis and hemostasis through the different receptors expressed on their surface, being involved in both detrimental and beneficial functions in the complex pathophysiology of ischemic stroke. Indeed, after an ischemic insult, platelets inside the hypoxic microvasculature affected by the ischemic event are exposed to the extracellular matrix where binding is initiated, subsequently leading to platelet activation, aggregation, and thrombi formation [12]. Additionally, platelets are able to interact with different types of immune cells inducing a thromboinflammatory crosstalk response [14] either positively contributing to angiogenic processes [15] and neovascularization, or promoting the secretion of pro-inflammatory soluble factors [16]. This ischemic scenario debilitates the blood-brain barrier (BBB) allowing immune cells to infiltrate the infarcted area affecting neuronal homeostasis and microglial activity, and subsequently promoting the development of the infarct lesion. Additional immune cells may also extravasate from perivascular areas, leptomeningeal spaces and the choroid plexus into the ischemic brain also exacerbating cerebral injury [17]. Importantly, depending on the immune cell type involved, i.e., neutrophils, monocytes, and T or B cells, their dynamic contribution to tissue damage or regeneration strongly differ over time.

Thus, we here provide an updated overview of the role of thromboinflammation in stroke pathomechanism centered on platelet-mediated microvascular impairment, cell-specific neuroinflammatory response, and the associated cellular crosstalk. A broad overview of the most relevant rodent transgenic lines is extensively presented encouraging novel research perspectives and further preclinical investigation [13]. Additionally, we thoroughly reviewed thromboinflammation-related therapies currently under clinical assessment, presenting an up-to-date overview of the therapeutic advances in the field.

## Thrombosis and inflammation: the concept of thromboinflammation

Despite a fast restoration of cerebral blood flow after stroke, recanalization of the occluded vessel entails progressive tissue damage, also known as ischemia-reperfusion injury. Brain reperfusion triggers a complex pathomechanistic cascade of thrombotic factors and secondary thrombotic events deeply involved in vascular lesions after stroke promoting platelet activation and thrombi formation. Similarly, cerebral reperfusion elicits a strong inflammatory response including the upregulation of cell adhesion molecules, cytokines release, and transmigration of several subsets of leukocytes, altogether mediating neuroinflammation [18]. This complex interplay between inflammation and coagulation processes, so-called thromboinflammation, is a key contributor to post-stroke tissue damage strongly associated with neuronal death and vascular dysfunction [19].

### Thromboinflammation and the kallikrein/kinin system

**Fig. 1:**
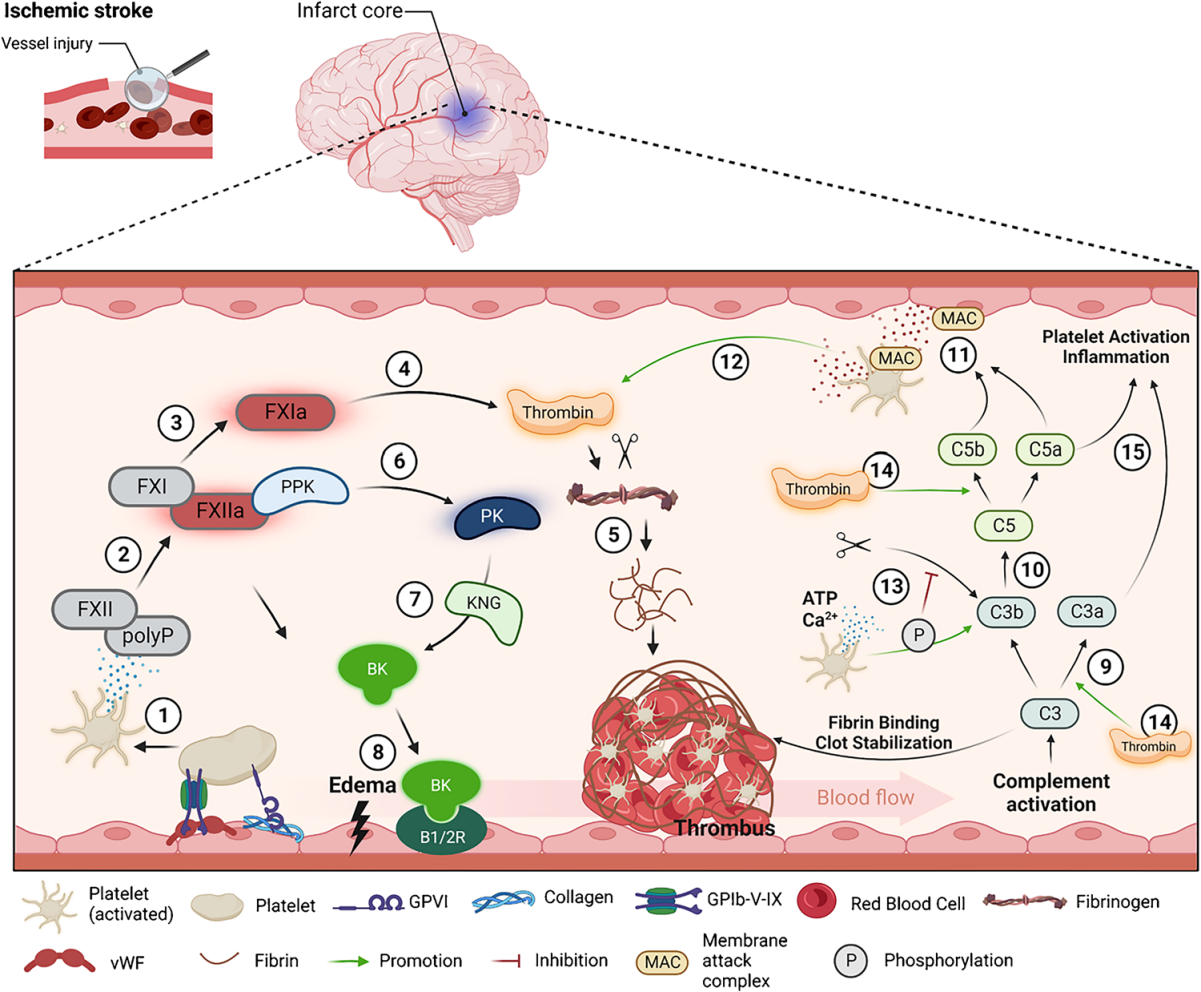
The role of the plasma kallikrein-kinin system on thromboinflammation. (1) Upon endothelial damage, platelets are activated through the binding of the GPIb-V-IX to vWF, or GPVI to exposed collagen. (2) Activated platelets release polyphosphates which activate FXII. (3) FXIIa activates FXI, (4) leading to the subsequent activation of thrombin. (5) Thrombin facilitates the enzymatic cleavage of fibrinogen to fibrin, forming and stabilizing the thrombus. (6) The contact-kinin pathway itself is initiated by FXIIa, which cleaves PPK into PK. (7) PK subsequently acts on KNG, inducing the release of the proinflammatory peptide hormone BK. (8) Consequently, BK binding to its endothelial bradykinin receptor 1 and 2 initiates several inflammatory signaling cascades resulting in breakdown of tight junction proteins and edema formation. (9) Activation of the complement system leads to cleavage of the key molecule C3 into C3a and C3b. (10) Subsequently, C5 is cleaved into C5a and C5b, (11) resulting in the formation of the MAC on endothelial cells and platelets. (12) The MAC induces the release of nanoparticles that enhance the proteolytic formation of thrombin by FVa, initiating coagulation. (13) In parallel, large amounts of ATP and Ca^2+^ are released which phosphorylate C3b and attenuates proteolytic cleavage of C3b. (14) Thrombin also promotes the conversion of C3 and C5 to their effector molecules (C3a, C3b, C5a, C5b) and promotes complement activation. (15) Subsequently, C3a and C5a are capable of enhancing the inflammatory response as well as platelet activation. Abbreviations: ATP, adenosinetriphosphat; B1/2R, bradykinin receptor 1/2; FXII, factor XII; GP, Glycoprotein; KNG, high-molecular-weight-kininogen; MAC, membrane attack complex; P, phosphorylation; PK, plasma kallikrein; polyP, polyphosphates; PPK, plasma prekallikrein; vWF, von Willebrand factor. Created with BioRender.com

The contact system, also named plasma kallikrein-kinin system (KKS), consists of a network of serially connected serine proteases mediating thrombus formation, vascular permeability, and blood pressure changes upon brain ischemia. The contact-kinin pathway is initiated by the activation of the blood coagulation factor XII (FXIIa), which cleaves plasma prekallikrein into plasma kallikrein (PK). PK subsequently acts on high-molecular-weight kininogen, inducing the release of the proinflammatory peptide hormone bradykinin (BK) [20]. Consequently, BK binding to its endothelial bradykinin receptor 1 and 2 (B1R/B2R) initiates several inflammatory signaling cascades resulting in thrombin generation and the influx of circulating immune cells, altogether resulting in thrombus formation and neuroinflammation [21, 22] (Fig. 1). Among the different KKS components, PK represents one of the most promising therapeutic targets due to its dual mode of action as a proinflammatory and prothrombotic enzyme. Indeed, genetic deletion of plasma kallikrein [22], kininogen [23] or bradykinin receptor 1 [21], together with the pharmacological blockade of plasma kallikrein [21, 22] significantly mitigated intracerebral thrombus formation and stabilized the blood-brain barrier, thereby reducing the number of brain-infiltrating immune cells [22, 24].

In addition to cleaving kininogen, PK also induces adenosine diphosphate (ADP)-dependent platelet aggregation after interacting with the surface of platelets through binding to αIIbβ3 integrin and cleaving protease-activated-receptor (PAR)-1 [25]. Moreover, the KKS is also able to interact with the thrombolytic agent rt-PA, frequently administered to stroke patients to promote clot dissolution. Indeed, elevated bradykinin levels and an increase in cleaved kininogen could be observed in stroke patients after rt-PA infusion [26]. In FXII^-/-^ or PK^-/-^ mice, kininogen was not altered after rt-PA administration demonstrating that rt-PA-mediated kininogen cleavage requires PK and FXII action. Therefore, either genetic deficiency or pharmacological inhibition of these mediators reduces rt-PA-dependent intracerebral hemorrhage, edema formation, and infarct volume upon ischemic stroke, supporting the hypothesis that FXII activates plasma kallikrein upon rt-PA treatment [26, 27].

### Thromboinflammation on microcirculation and blood-brain barrier leakage

Brain microvasculature (<100μm of diameter) includes the vast majority of the endothelial cells (EC) present in the central nervous system (CNS) providing all metabolic and nutrition requirements to the brain parenchyma, also supporting several neurovascular physiological functions [28]. Under physiological conditions, Ecs maintain vascular integrity by exerting an anti-platelet, anti-coagulant, and anti-inflammatory role. Indeed, quiescent Ecs release potent platelet antagonists including the cluster of differentiation (CD) 39/ecto-adenosine diphosphatase enzyme and prostaglandin I2 (PGI2) preventing platelet adhesion and activation. Moreover, basal EC-dependent nitric oxide release prevents leukocyte recruitment in the vessel wall via minimizing P-selectin expression, blocking chemokine expression, and reducing transcription of adhesion molecules, such as E-selectin, vascular cell adhesion molecule (VCAM)-1, and intercellular adhesion molecule-1 (ICAM-1). Similarly, PGI2-dependent reduction of leukocyte adhesion and activation directly down-regulates inflammation [29].

Upon an ischemic event, the release of unstable free radicals and specific matrix metalloproteinases (MMP) debilitates endothelial tight junctions damaging the neurovascular unit, altogether compromising microvascular integrity and increasing BBB permeability [30]. This microvascular homeostasis dysfunction directly promotes major endothelial dysregulation within the close interaction amongst brain microvascular endothelial cells, leukocytes, platelets, and erythrocytes; thus promoting thrombosis and neuroinflammatory events [31]. Certainly, neutrophils are the first-in-line defensive cells, rapidly recruited within minutes and later transmigrated through the endothelium upon ischemic conditions. This neutrophil influx promotes a pro-thrombotic cascade increasing the expression of selectins (P- and E-selectin) and the adhesion molecule ICAM-1, thus resulting in neurovascular microthrombosis. Similarly, neutrophils can express several pro-neuroinflammatory mediators including neutrophil extracellular traps (NETs), reactive oxygen species (ROS), and serine proteases which collectively enhance thromboinflammation [28]. Platelets, apart from being first responders to endothelial disruption, belated neuroinflammatory events are also able to initiate a platelet-dependent response, ultimately leading to thrombin formation and fibrin crosslinks via diverse glycoproteins, activation of tissue factor (TF) receptors and selectins, and promoting macrophage (Mac)-1/fibrin interaction [18]. Thus, the direct crosstalk among various cell types, such as microvascular endothelial cells, platelets, neutrophils and other immune cell types is heavily involved in thromboinflammatory processes following the ischemic insult.

## The role of platelets in thromboinflammation

### Platelet activation

Platelets are known to play both beneficial and detrimental roles after stroke. Indeed, the ability of platelets to contribute to inflammation, immunity, angiogenesis, and neurogenesis remains, however, partially controversial [15, 16, 32–36]. Although their main purpose is to maintain vascular integrity, under pathological conditions such as thrombocythemia or thrombocytosis, platelets are able to promote excessive thrombus formation or excessive bleeding [37]. Thus, interfering with the coagulation cascade carries both therapeutic opportunities as well as significant risks.

The three main glycoprotein receptors (GP) present on the platelet surface are GPIb, GPVI, and GPIIb/IIIa whereas each of those inherits specific functions resulting in thrombus formation (Fig. 1). After the ischemic event, platelets are exposed to the extracellular matrix and binding is initiated by the common receptor complex GPIb-V-IX. This contact is facilitated by the receptor subunit GPIbα coupling to the von Willebrand factor (vWF), which is either released by endothelial cells or platelets α-granules [38–40]. However, even though vWF is immobilized at the sites of vascular injury, the contact itself is happening quite rapidly only allowing platelets to tether, which is not sufficient to form a stable adhesion under high shear conditions. Importantly, both genetic deletion of the vWF [41] and pharmacological blockade of the respective binding site [42, 43] significantly reduce infarct volumes in experimental stroke models.

Still, this rolling of platelets is crucial to activating other receptors, for instance, GPVI to interact with their respective binding partners, i.e., collagen, fibrin, and laminin [44–46]. Specifically, GPVI is known to be the major signaling receptor for collagen, leading to the immobilization of platelets and coagulation via direct modulation of FXII. Activation through fibrin, instead, promotes and accelerates thrombus growth as well as stabilization; while the activation of GPVI via laminin induces spreading and adhesion of platelets, although the exact role in physiological and especially pathophysiological conditions is yet to be elucidated. Importantly, GPVI is not able to maintain a stable adhesion between platelets but instead, induces potent platelet activation. Mediated by a tyrosine phosphorylation cascade, the activation leads to shape changes, the release of granule-stored factors, and intracellular calcium mobilization [44, 47–49], altogether resulting in the release of secondary mediators such as ADP and thromboxane. Ongoing GPVI signaling not only results in the release of procoagulant and proinflammatory factors, but also, together with platelet derived polyphosphates, leads to the activation of FXII which subsequently triggers the intrinsic coagulation cascade and the contact system [50, 51]. Indeed, pharmacological blocking [52] or genetic depletion of GPVI [53] and FXII [51] reduces infarct development by preventing platelet adhesion and aggregation, thereby directly modulating thromboinflammation [21, 22, 51, 54]. Additionally, thrombi formation through platelet aggregation requires a scaled platelet response including conformational changes of the GPIIb/IIIa receptor through high affinity binding to its partners such as fibrinogen [55], to finally stabilize the platelet clot. Contrary to the abovementioned receptors, modulation of GPIIb/IIIa signaling in experimental stroke models increased intracerebral hemorrhage while reducing overall survival [53, 56]. Clinically, trials evaluating the effect of GPIIb/IIIa inhibitors have been terminated prematurely due to dramatic bleeding events [57, 58].

During thrombus formation and after restoration of cerebral blood flow, the complement system in conjunction with platelets is activated [59] enhancing cerebral damage [60]. The central molecule complement component 3 (C3) connects the classical, lectin, and alternative complement pathways. In detail, the activation of the complement system leads to C3 and C5 being cleaved into their respective bioactive fragments C3a, C3b and C5a, C5b. During thrombosis, C3a and C5a foster platelet activation and aggregation by inducing exposure of P-selectin for the recruitment of neutrophils to the endothelium [61]. Precisely, C5a induces upregulation of TF and plasminogen activator inhibitor-1 expression on neutrophils, monocytes and endothelial cells [62–64]. C5b also supports the formation of the membrane attack complex (MAC) which initiates coagulation and influences platelet activation [65]. MAC can subsequently induce the release of membrane microparticles from platelets and endothelial cells that expose additional binding sites for Fva leading to enhanced prothrombinase activity and coagulation [66, 67]. Later, thrombin is able to cleave C3 and C5 into their respective fragments C3a, C3b, C5a, and C5b resulting in increased complement activation as part of a positive feedback loop [68]. Experiments carried out in ischemic animal models indicate that the blockade of complement components exerts protective effects, including inhibition of C1, C3, C5 and the MAC [69–72]. Translationally, C3 and C3a levels have been shown to be upregulated in plasma from ischemic stroke patients [73], similar to complement deposits in human post-stroke brain tissue [74].

### Platelet function in cerebral microvascular impairment

Besides their hemostatic actions, platelets directly act on the microvasculature to maintain vascular integrity, preserve microvascular function, and closely interact with the surrounding immune cells. Under ischemic conditions, the microvasculature develops an inflammatory phenotype linked to impaired blood flow regulation and recruitment of inflammatory cells, ultimately leading to the leaking of vascular barriers [31, 75, 76]. Platelets, in this scenario, not only seal breaches in the endothelial wall but also prevent leukocyte infiltration by tightening the endothelial belt around transmigrating leukocytes, preventing hemorrhage and plasma protein extravasation into the surrounding tissue [77]. Additionally, platelet adhesion and clotting at sites of endothelial injury during inflammatory processes directly prevent hemorrhages [78]. In fact, platelets exert a variety of processes including activation upon contact with damaged endothelium and exposed collagen as previously described. Moreover, even though the evidence is scarce, there are also hints that platelets are activated by contact with intact endothelium in the microcirculation. Specifically, degranulation induces disruption of endothelial cells consequently attracting and activating large amounts of additional platelets [79, 80]. Typical anti-platelet agents were unable to tackle this prothrombotic and potentially risk-bearing phenotype [81]. Thus, it is worth highlighting the role of intact platelets in maintaining microvascular integrity.

### Platelets in neovascularization

The relevance of platelets in facilitating stroke recovery after an ischemic event is still questionable [12]. Platelets store and release considerable amounts of trophic factors including the platelet-derived growth factor (PDGF), vascular endothelial growth factor, fibroblast growth factor, transforming growth factor (TGF)-β, platelet factor 4 (PF4), and the brain-derived neurotrophic factor, all playing a major role in cerebral vascularization [82, 83]. These elements are able to orchestrate the subsequent response to ischemia promoting neurogenesis and neovascularization from 12 hours up to 21 days after the ischemic event [84]. Indeed, using platelet lysate as treatment significantly increases new blood vessels and newborn cells surrounding the ischemic lesion, even though some of them remain undifferentiated [34, 35, 85]. Enhancing angiogenic and neurogenic processes by platelet lysate treatment also contributed to decreased behavioral deficits up to 90 days after stroke [35]. However, in other brain ischemia models, platelets remain as regulators of neovascularization either in a positive or negative direction, suggesting a sophisticated time-dependent role within stroke pathophysiology [15, 16]. Promoting angiogenic processes in boundary regions around the ischemic core leads to improved behavioral performance [86]. Importantly, the role of platelets in neovascularization and neurogenesis still remains poorly described since the majority of post-stroke studies tackling thromboinflammation are focused on acute experimental settings frequently overlooking long-term scenarios. Thus, highlighting and designing future experimentation based on recent findings could shed a new light on platelets as key regulators of different thromboinflammatory processes, carrying out important roles in stroke pathophysiology and recovery beyond the hyper-acute phase.

## Neuroinflammatory response after ischemic stroke

**Fig. 2:**
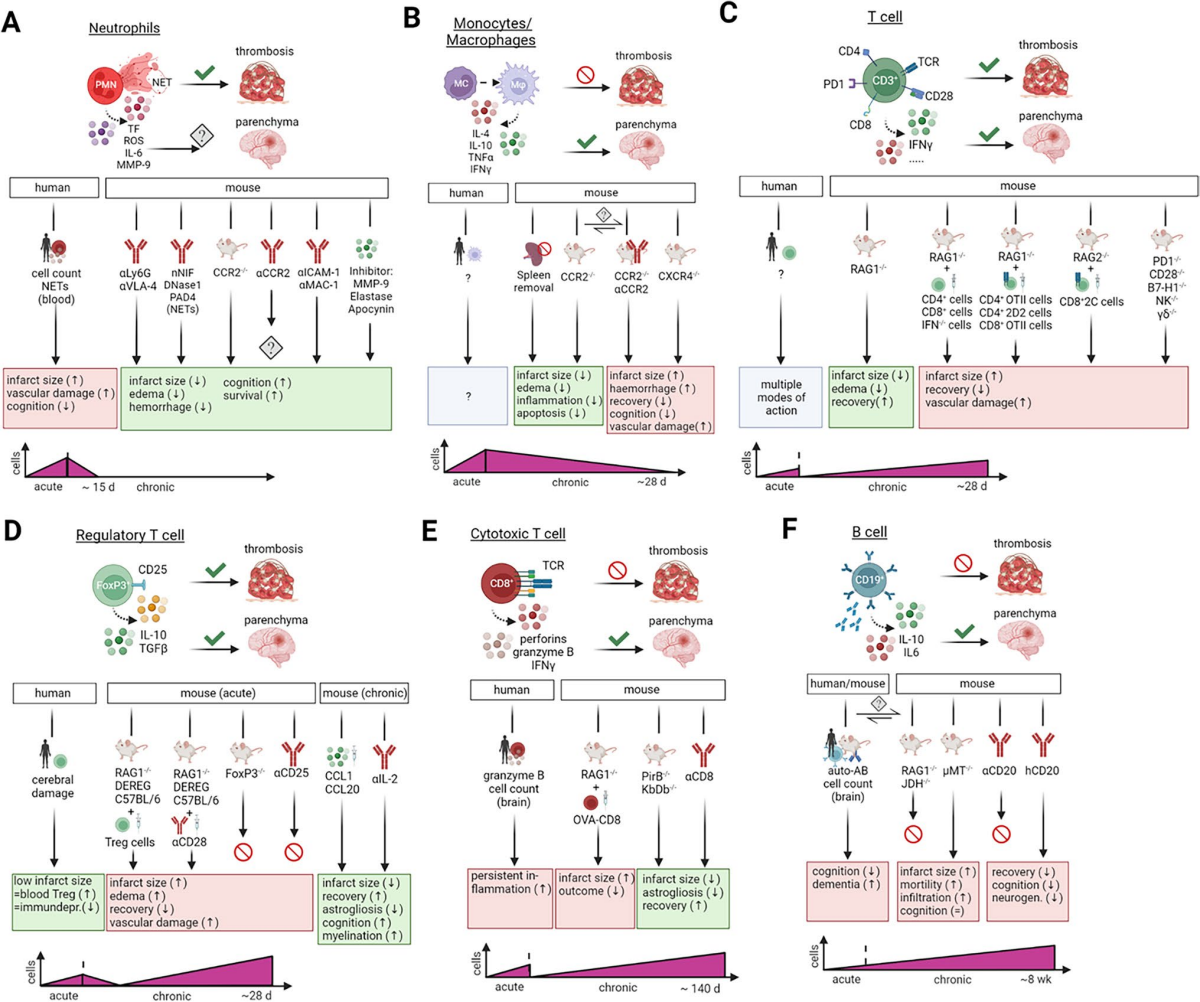
The role of immune cells in thrombosis and tissue damage after ischemic stroke. (**A**) During acute ischemic stroke, neutrophils promote thrombosis via release of NETs, inflammatory factors and BBB destabilizing factors. Influx of neutrophils into the brain parenchyma heavily depends on reperfusion and is still controversially discussed regarding their role in persistent tissue damage and repair. In humans, neutrophil cell count and NET propagation in the peripheral blood are connected to severe ischemic stroke. Depletion of NETs, neutrophils and blocking of adhesion to the endothelial barrier in mice correlated with improved ischemic outcome. Early inhibition of neutrophil-dependent mediators of damage e.g., MMPs and ROS result in enhanced cerebral protection. (**B**) The role of MM in acute ischemic stroke is not fully understood. Yet, MM are not involved in thrombosis formation, but monocytes are able to differentiate upon thrombus formation into inflammatory macrophages. Influx into the brain parenchyma afterwards might shape the inflammatory milieu found in ischemic lesions. Absence of MM, either through splenectomy or CCR2 deletion, led to less stroke burden in mice. However, CCR2 seems to be controversial since other studies showed enhancement of detrimental stroke processes. Accordingly, interrupting the chemokine axis for macrophage migration into the brain results in severe ischemic stroke. (**C**) General stroke experimentation with T cells uncovered that lymphocytes are involved in thrombus formation and immunological responses in the brain parenchyma. T cell deficiency led to protection against severe consequences of ischemic stroke. Genetic alteration in TCRs of T helper and cytotoxic T cells claimed TCR independent inflammation during the acute phase of ischemic stroke. (**D**) Knock-out of natural killer cells or conservative γδ T cells and deletion of co-stimulatory signal molecules further underlines a general independent inflammatory response after acute ischemic stroke. Tregs are an anti-inflammatory subset of T helper cells. In stroke patients, the number of circulating Treg cells positively correlates with the infarct size and absence of immunosuppression. Experimentation with mice, highlighted a detrimental role of Tregs participating in thrombus formation leading to severe ischemic damage and worse functional outcome. Adoptive transfer of Tregs into specific T cell deficient models or expansion of the Treg population was detrimental while transcription factor knock-out models and antibody depletion experiments targeting Tregs were inconclusive. In the chronic phase, however, Tregs are mandatory for recovery in the chronic phase after stroke proven in different experimental settings. (**E**) CTLs exert their surveillance function via TCR mediated apoptosis induction and secretion of inflammatory cytokines. CTLs are not involved in thrombosis propagation. Despite CTLs mediate detrimental effects via TCRs in the chronic phase, in the acute ischemic stroke however, CTLs enhance inflammation in a TCR-independent manner. Depletion of CD8^+^ T cells or use of knock-out models for TCRs of CTL led to stroke protection even in the chronic phase after ischemic insult. In patients biopsies, the number of CTL and the amount of granzyme B in brain parenchyma were in accordance with the severity of ischemic brain damage. (**F**) B cells are ambivalent immune cells in the pathogenesis of ischemic stroke. In thrombosis development, B cells have been extensively investigated in mice and found negligible for stroke induction. However, knock-out mouse models found no effects (RAG1^-/-^; JHD^-/-^) or beneficial effects (μMT) of B cells on brain protection processes in the acute phase of ischemic stroke. Antibody-mediated depletion of B cells controversial mediated cognitive decline and aggravated recovery in the chronic phase after stroke. Experiments in mice and humans led to contrary results. Abbreviations: μMt, inactivating mutation in the first M transmembrane exon of the μ heavy chain domain; BBB, blood-brain barrier; CCL, chemokine (C-C motif) ligand; CCR, chemokine (C-C motif) receptor; CTL, cytotoxic T cell; CXCR, chemokine (C-X-C motif) receptor; JHD, JH gene deficiency for antibody heavy chain; KbDb, gene of the major histocompatibility complex class; MC, monocyte; MM, monocytes/macrophages; MMP, matrix-metalloproteinases; Mφ, macrophage; NET, neutrophil extracellular trap; PirB, paired immunoglobulin-like receptor B; PMN, polymorphonuclear leukocytes; RAG, recombination activating gene; ROS, reactive oxygen species; TCR, T cell receptor; TF, tissue factor; Treg; regulatory T cells; VLA, very late antigen. Created with BioRender.com

Thromboinflammation involves the progression of the coagulation cascade and the activation of immune cells at the thrombus site, thus leading to blood-brain barrier damage. This scenario results in the CNS opening towards the periphery allowing immune cells to enter the infarcted area and initiate several immunological processes responsible for secondary infarct growth. Depending on the type of immune cell involved, their contribution to tissue damage or regeneration strongly differs. Therefore, deep understanding of this complex immunological network, incorporation of recent findings, and their accurate interpretation are key to develop mechanistic insights into ischemic stroke pathology (Fig 2).

### Neutrophils

The cell number of neutrophil granulocytes in circulation increases ~4-6h after the ischemic event resulting in direct migration into the infarct area within 6-8h after the ischemic event (Fig. 2A) [87]. Accumulation in the damaged area peaks 1-3 days after the event, being no longer detectable after 15 days [88]. During ischemic brain injury, a plethora of chemo- and cytokines initiate the recruitment and activation of neutrophil granulocytes. Specifically, chemokine (C-X-C motif) ligand (CXCL)-1,2 and 5 and their respective receptors (CXCR)-1,2 and 4 contribute to their recruitment within the ischemic brain lesion [89]. Indeed, the elevation of CXCL-1 and CXCL-5 was confirmed in cerebrospinal fluid from stroke patients together with the increased presence of chemokine (C-C motif) ligands 2, 3, and 5 in blood, mediating neutrophil recruitment [90]. In fact, induction of transient middle cerebral artery occlusion (tMCAO) in CCR2 knockout mice has a cerebroprotective effect due to reduced neutrophil infiltration post-stroke. However, using CCR2 as a therapeutic strategy turns out to be widely unspecific for neutrophils [91]. Contrary, targeting the neutrophil adhesion to the endothelial barrier by blockade of ICAM-1[92], Mac-1 [93], and selectins [94] have been shown as promising targets for improving post-stroke outcomes in rodents. These results, however, failed to be translated to stroke patients [95–97].

Preclinically, blocking neutrophil influx into the brain improves neurological outcomes in mice [98]. Specifically, neutrophil depletion reduces tissue damage, infarct volume, edema formation, and hemorrhagic transformation in different stroke animal models [98–100]. However, it seems controversial whether neutrophils reach damaged brain parenchyma after the ischemic insult since only a persistent accumulation in perivascular spaces around venules has been detected [87]. In fact, a study of post-mortem tissues of 16 ischemic stroke patients confirmed neutrophils accumulated in the leptomeninges and perivascular spaces, although very low numbers were found in the parenchyma [101]. Therefore, reperfusion appears to be critical for neutrophils to reach the injured brain parenchyma. Otherwise, without reperfusion, neutrophils will only reach the injured areas via retrograde collateral pathways from the leptomeningeal vessels to the perivascular space and then to the parenchyma [102, 103]. However, despite a low presence in the ischemic brain parenchyma, neutrophils critically contribute to vascular damage promoting thromboinflammation. Importantly, higher neutrophil counts in blood were associated with larger infarct volumes and poor clinical outcomes in stroke patients [101, 104, 105]. Thus, among others, neutrophils produce extracellular traps, tissue factors, and proinflammatory molecules that lead to severe BBB leakage and enhanced thrombus formation [106–110]. Additionally, targeting neutrophil-mediated mechanisms that disrupt BBB integrity through the secretion of proteases (matrix metalloproteinases, elastase, cathepsin G, and proteinase 3) turned out to protect mice from severe ischemic injury in several preclinical stroke models (Fig. 2) [87, 111].

### Monocytes/macrophages

Infiltration of monocytes/macrophages (MM) can be detected within 24h post-stroke reaching its peak latest at day 3 after the ischemic event, although they can still be detected in the ischemic area up to 28 days while they undergo phenotype changes (Fig. 2B) [106, 112, 113]. Upon reperfusion, monocytes infiltrate into the infarct area, primarily within the core and peri-infarct area [114]. Among other inflammatory cytokines, adhesion molecules, and chemokines, monocyte infiltration is largely dependent on CCR2, directly linked to the monocyte chemoattractant protein 1 [115, 116]. In fact, during acute ischemic stroke, CCR2-deficient mice developed smaller infarct volume, reduced brain edema, and attenuated expression of inflammatory molecules [91, 117]. In line with this, splenectomy or splenic irradiation of MM led to decreased MM infiltration into the ischemic hemisphere together with reduced infarct volume, neuronal apoptosis, BBB damage, and cerebral edema [91, 118, 119]. Other studies, however, demonstrated that the absence of MMs via CCR2 knockout or CCR2 antagonist treatment exacerbated brain lesions and elevated the risk of hemorrhagic transformation [120, 121]. Blocking the infiltration of macrophages also impaired spontaneous long-term functional recovery and aggravated neurological dysfunction [122]. However, inhibition of CCR2 or direct splenectomy reduced monocyte infiltration into the cerebral ischemic tissue not affecting infarct size nor neurological recovery [118, 123, 124]. These experimental contradictions may be caused by the diversity of (i) experimental animal models used, (ii) the severity of ischemia, and (iii) the different methods used to inhibit the entry of monocytes into the ischemic tissue. Additionally, the phenotype of MMs, i.e., M1 as a pro-inflammatory role and M2 associated with inflammatory resolution and repair, definitely play a critical role in ischemic pathomechanisms. In mice after tMCAO, M2 markers are increased within the first weeks after occlusion and decrease thereafter, suggesting a persistent proinflammatory stimulus that destabilizes a permanent M2 phenotype switch in the ischemic brain lesion [113, 121, 125]. In contrast, studies in human patients reported an initial M1 inflammatory phenotype within the lesion that shifts to anti-inflammatory M2 features as the lesion matures [101]. Overall, the role of MM and its effects on stroke pathophysiology is highly controversial since adverse effects cannot be fully attributed to a single MM phenotype or to an MM-mediated pathomechanism. However, it is likely that MMs play supportive roles in inflammation and repair upon stroke.

### T cells

Upon stroke, the hallmarks of thromboinflammation are evidently centered on microvascular dysfunction and a remarkable T cell-dependent increased inflammatory response subsequently leading to tissue death (Fig. 2C). CD3^+^ T cells are present in the ischemic area as early as day 1 remaining in high numbers until 3–5 days after tMCAO [126, 127]. Moreover, despite most studies being limited to the first 7 days post-stroke, a long-term T cell response can be demonstrated in the brain parenchyma up to day 28 [128, 129]. During thromboinflammation, T cells exert their mechanistic contribution by interacting with the endothelium via the very late antigen-4:ICAM-1, and the lymphocyte function-associated antigen-1:ICAM-1. Similarly, both endothelium and platelets modulate T cells via P-selectin glycoprotein ligand-1 (PSGL)-1:P-selectin, CD40:CD40L, or through CD84 (only platelets) [130]. These interactions cause microvascular dysfunction, increased thrombus formation and, subsequently, impaired cerebral reperfusion after tMCAO. For instance, soluble CD84 accumulates in the microvessels, binds to CD4 T cells, and promotes T cell recruitment [14]. Then, T cells accumulate in small cerebral vessels that interact with the endothelium promoting reperfusion injury, and therefore, stroke progression [131]. Significant reductions in leukocyte infiltration and platelet adhesion have been described in CD4^+^ T cell^-/-^, CD8^+^ T cell^-/-^, Interferon (IFN)-gamma^-/-^, and recombination activating gene (Rag) 1^-/-^ stroke mice. Indeed, immunodeficient Rag1^-/-^ mice lacking both T and B cells show a significantly reduced infarct volume and improved neurological outcome. Indeed, when these animals were reconstituted with wild-type CD4^+^ and CD8^+^ T lymphocytes or IFN-gamma^-/-^ splenocytes, the detrimental phenotype was rescued. Thus, T cells contribute critically to cerebral ischemia, although their deleterious effect does not depend on antigen recognition, T cell receptor (TCR) costimulation, or thrombus formation. TCR-transgenic mice carrying a single CD8^+^ (transgene receptor of cytotoxic T cells (2C)/RAG2, deficient in recombination activation gene (OT)II/RAG1 mice) or CD4^+^ TCR (OTII/RAG1, 2D2/RAG1 mice), and mice lacking costimulatory TCR signaling (programmed cell death protein (PD)1^-/-^, CD28^-/-^, PD1^-/-^, B7-H1^-/-^ mice) were highly susceptible to tMCAO. Similarly, genetic deletion of natural killer T cell and γδ-T cells seem not to protect from severe cerebral damage [132]. Altogether, the role of different T cell subsets in ischemic stroke appears to be both time- and severity-dependent.

### Regulatory T cells (Tregs)

As a subset of T helper cells, Tregs mediate suppressive and tolerogenic functions limiting autoimmunity (Fig. 2D) [133]. In stroke, however, the dynamics of Tregs and their pathogenic role are highly time-dependent. Focusing on thromboinflammation, stroke volume was remarkably reduced in Tregs-depleted mice (DEREG) 1 day after the stroke insult, while 3 weeks of reconstitution treatment in DEREG mice completely reverted the detrimental phenotype leading to enlarged infarct sizes [134]. Similarly, injecting CD4^+^CD25^+^ Treg cells 1 day before tMCAO induction resulted in enhanced infarct size; while the transfer of Treg cells into RAG1^-/-^ mice 1 day after tMCAO confirmed the detrimental role of regulatory T cells during acute ischemic stroke [134]. Likewise, expansion of Tregs via anti-CD28 superagonist (CD28 SA) to C57BL/6, RAG1 and DEREG mice results in larger infarct volumes and worse neurological outcome 1 day after tMCAO [135]. In related studies, despite reporting a decreased infarct size after CD28 SA injection, pronounced functional deficits were visible 7 days after stroke [135]. Importantly, Treg activity could be directly modulated by interleukin (IL) release. Indeed, intraperitoneal application of IL-2/IL-2 antibodies prior to tMCAO increased the number of Treg cells, associated with a decreased infarct size and improved sensorimotor outcomes [136, 137].

Within the sub-acute ischemic phase, deletion of Tregs cells using anti-CD25 antibodies 3 or 14 days after tMCAO neither changed the infarct volume nor the behavioral outcome at 14 and 27 days [138]. Similarly, Treg depletion starting 7 days after ischemic stroke did not alter primary outcome parameters and neuronal tissue loss in the later stages. However, concerning long-term stroke recovery, Treg depletion significantly worsened later neurological outcomes suggesting the important role of this cell type in neuro-repair during the chronic phase post-stroke [139, 140]. Long-term depletion of Tregs increases the number of reactive astrocytes and enhances the expression of neurotoxic genes [140] while preventing white matter injury repair through oligodendrogenesis. Similarly, depletion of Tregs causes a decrease in re-myelination, thinner myelin sheaths, reduced nerve fiber conduction from myelinated axons, and reduced numbers of newly-generated oligodendrocytes [139], all closely related to long-term recovery and tissue repair.

Clinical observation studies have indicated that infarct volume was associated with reduced T helper cell counts in the periphery between days 1-4 upon stroke [141]. However, despite the suppressive effects of Treg cells being attenuated, the proportion of Treg cells in the peripheral blood is known to be increased in ischemic patients compared to controls starting at day 7 and lasting until the 3rd week after the insult [142, 143]. However, different infarct volumes and diverse lesions in the hemisphere thus result in the different effects of Treg cells [144]. Altogether, it remains unclear how Treg functions modulate stroke pathophysiology in a highly dynamic and time-dependent manner after the ischemic event.

### Cytotoxic T cells

CD8^+^ T cells (CTL) mediate their cytotoxic effect by antigen-recognition through the T cell receptor/major histocompatibility complex-1 and pro-inflammatory cytokine secretion to eliminate viral infection or defective cells (Fig. 2E) [145]. In stroke, the deleterious effects of CD8^+^ T cells predominantly occur within the chronic phase rather than the first days after the occlusion although early depletion studies have also been conducted [127, 146]. Adoptive transfer of ovalbumin (OVA)-TCR CD8^+^ T cells resulted in reduced CD8^+^ T cell activation and infiltration, lowering infarct volume at day 7 and therefore suggesting opposing TCR-dependent effects [147]. Similarly, delayed depletion of CD8^+^ T cells at day 10 after ischemic stroke improves recovery and reduces long-term chronic neuroinflammation in male mice [148], also found at days 7 and 14 including a rescued phenotype in Rag^-/-^ mice after CTL reconstitution [146, 149]. Increased numbers of CD8^+^ T cells detected in the CNS also correlate with severe cerebral damage and worse functional outcome within the chronic phase after an ischemic event [148]. Clinically, robust infiltration of activated T cells into the infarct brain has been reported on day 140 after stroke, of which > 60% are CD3^+^ CD8^+^ T cells, suggesting a long-lasting T cell response in the human ischemic brain [101, 148, 150].

### B cells

As a subset of the adaptive immune system, B cells are able to induce a humoral immune response and lead antigen presentation (Fig. 2F) [151, 152]. In mice after experimental cerebral ischemia, B cells infiltrate into the brain starting after day 7 up to 12 weeks after tMCAO [153]. Infarct size remains unaffected in T and B cells deficient mice (RAG1^-/-^) confirming a non-detrimental role of B cells in ischemia [134]. Accordingly, other B cell-depletion models, i.e., anti-CD20 or JH gene deficiency for antibody heavy chain (JHD), showed no changes in stroke volumes or functional outcomes [154]. Contrary, B cells comprising an inactivating mutation in the first M transmembrane exon of the μ heavy chain domain (μMT) mice develop significantly larger lesions, higher mortality and elevated infiltration rates 48h after tMCAO; a phenotype fully rescued via reconstitution of B cells and directly attributed to IL-10-produced by B cells [155, 156]. In addition, polyclonal activated and non-activated B cell transfer into syngeneic mice ameliorates functional deficits and reduces inflammatory influx within the first 96h after stroke via IL-10–mediated up-regulation [157]. Both phenotypic scenarios could therefore be attributed to diminished IL-10-dependent recruitment of neutrophils into the brain resulting in less cerebral damage [158]. Clinically, B cells are directly associated with an increased risk of developing dementia and cognitive decline. Interestingly, B cell counts are significantly higher in patients suffering from post-stroke dementia [153, 159]. Thus, B cell diapedesis occurred in areas remote to the infarct mediate motor and cognitive recovery linked to cognitive prognosis [159]. Hence, whether the role of B cells is beneficial or detrimental has not yet been clearly described, although their relevance in thromboinflammation is of ultimate importance for disease progression.

## Interactions between platelets and immune cells

**Fig. 3:**
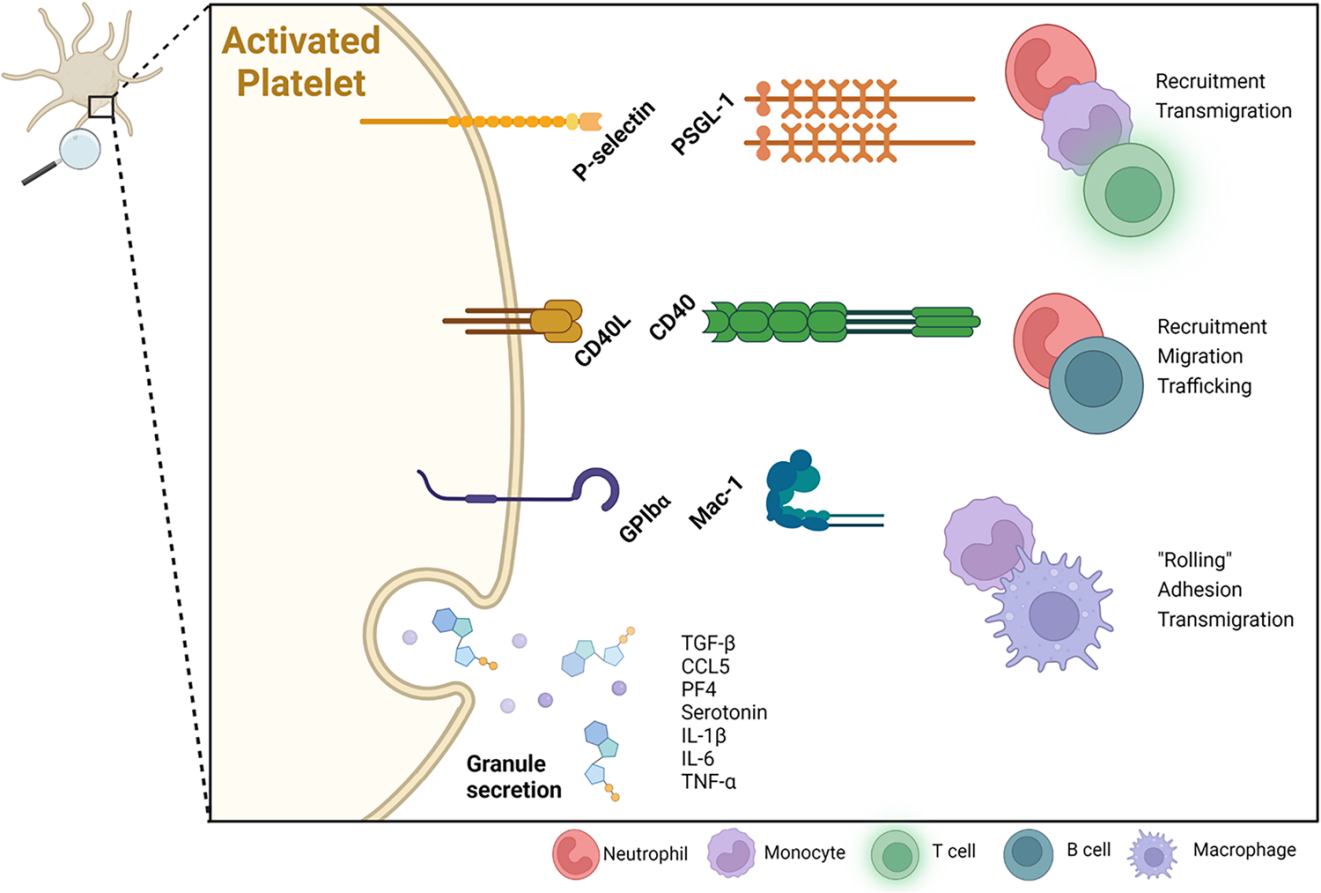
Interaction between platelets and immune cells. An intense cross-talk between platelets and immune cells takes place during ischemic stroke and the subsequent neuroinflammatory cascade. Platelets, especially after activation, express multiple receptors, enabling them to interact with immune cells in the peripheral blood. P-selectin on the platelet surface interacts with PSGL-1 on neutrophils, monocytes and activated T cells, leading to recruitment of additional immune cells followed by transmigration through membranes. CD40L mainly interacts with its correspondent receptor CD40 on neutrophils and B cells, resulting in recruitment, migration and trafficking of immune cells. The interaction between GPIbɑ and Mac-1 on monocytes and activated macrophages subsequently leads to a “rolling” behavior of the affected cells, facilitating adhesion to the endothelial surface as well as transmigration. Additionally, activated platelets secrete a plethora of signaling molecules including chemoattractants, chemokines and other inflammatory mediators. Abbreviations: CCL, chemokine (C-C motif) ligand; CD, cluster of differentiation; GP, glycoprotein; IL, interleukin; Mac-1, macrophage-1 antigen; PF, platelet factor; PSGL, P-selectin glycoprotein ligand; TGF, tumor growth factor; TNF, tumor necrosis factor. Created with BioRender.com

The intense crosstalk between platelets and a variety of immune cells highlights the relevance of this cell-cell interaction in stroke pathophysiology. Platelets can contribute to inflammation via the secretion of soluble factors such as (i) macrophage inflammatory protein 1α and CCL5 for the recruitment and activation of leukocytes, (ii) PF4 to recruit neutrophils, (iii) sCD40L to potentiate IgG production of B cells, and (iv) PDGF, IL-1β and TGF-β to modulate the inflammatory response [160–168]. Additionally, platelets are also able to take part in inflammatory processes by direct interaction with immune cells through multiple surface receptors and ligands, primarily GPIbα, P-selectin, and CD40L [169–171] (Fig. 3).

### GPIbα

GPIbα, as part of the GPIb-V-IX receptor complex, is responsible for binding to vWF and also known to bind to the Mac-1 antigen on leukocytes promoting vascular inflammation and thrombosis [172]. Upon activation, leukocyte Mac-1 can bind to endothelial ICAM-1 to support transmigration followed by binding of ICAM-2 present on platelets to leukocyte Mac-1 [173]. Blocking either GPIb or its ligand vWF A1 domain with an antibody, or through genetic deletion, significantly reduced the number of infiltrated T cells as well as the overall infarct volume [43, 174, 175]. Overall, GPIbα is the most important receptor when it comes to platelet-neutrophil interactions, enabling a strong connection between these two cell types and the damaged endothelium. Subsequently, this connection facilitates neutrophils to transmigrate through the endothelium and promotes downstream signaling for the recruitment and adhesion of additional cells.

### P-selectin

P-selectin, also called CD62, is a glycoprotein receptor stored in α-granules which is upregulated and translocated to the surface upon platelet activation [176, 177]. The PSGL-1 on leukocytes acts as the counterpart mediating neutrophil rolling, and subsequently leading to leukocyte activation via Mac-1. The interaction between platelets and neutrophils also leads to transendothelial migration of neutrophils and the promotion of tissue inflammation [178, 179]. Thus, platelets are able to orchestrate the inflammatory response in different disease pathomechanisms by the formation of platelet-leukocyte-aggregates (PLA) at the site of injury, and recruitment of additional immune cells [180, 181]. Furthermore, this interaction is stimulated by binding of platelet P-selectin to leukocyte PSGL-1, followed by the interaction between GPIbα on the platelet surface and Mac-1 present on leukocytes to further stabilize the adhesion of PLAs [181, 182]. P-selectin deficient mice display fewer atherosclerotic lesions with a smaller number of leukocytes present in these lesions [183–185]. In line with these findings, clinical studies observed increased P-selectin expressions as well as high levels of PLAs in blood samples from stroke patients [186].

Activated platelets also induce monocyte production of cyclooxygenase 2, an enzyme responsible for the synthesis of other proinflammatory factors, which was found to be highly upregulated in chronic inflammatory conditions including brain ischemia [187, 188]. Additionally, the interaction of platelets with monocytes leads to the production and release of platelet-produced IL-1β, IL-6 as well as tumor necrosis factor α. These cytokines are strongly associated with leukocyte activation and increase the expression of several other proinflammatory mediators upon stroke [168, 189].

### CD40L

The receptor CD40L, mainly located on the surface of activated T cells, has also been found to be present on the platelet surface [190, 191]. Its counterpart, CD40, is expressed on a variety of cells, including B cells and neutrophils, and involved in the regulation of adaptive immunity [192]. After being cleaved via metalloproteinases, CD40L can be found in solution as sCD40L, acting as a modulator of inflammation [193], and subsequently binding to endothelial CD40 thus increasing the expression of multiple adhesion molecules (e.g., ICAM-1, VCAM-1) required for leukocyte migration and trafficking [194]. Blocking CD40L on platelets may preserve and enhance Treg response by promoting proliferation of forkhead-box-protein P3^+^ T cells [195]. Their differentiation requires high levels of TGF-β, as those released by platelets [196]. The amplification of Tregs via a super-agonist worsened stroke outcome, highlighting the participation of Tregs in lesion growth [135]. Also, adoptive transfer of Tregs to Rag^-/-^ mice, lacking mature T and B cells, resulted in an increased infarct size dependent on the presence of platelets. The presence of both Tregs and platelets reduced cerebral blood flow and increased brain fibrin levels compared to platelet-depleted mice, while the Treg immunological function remained unaltered, indicative of platelet-Treg-associated microvascular dysfunction and thrombosis [134]. Another platelet-born pro-infiltration factor is CD84, a soluble homophilic immunoreceptor that is shed from platelets after ischemic stroke. Mice deficient for platelet or T cell CD84 developed smaller infarct volumes and displayed fewer infiltrated T cells inside the brain parenchyma. Notably, higher CD84 levels on platelets isolated from stroke patients were associated with worsened clinical outcomes. According to this, soluble CD84 stimulates T cells and shapes their migratory behavior after cerebral ischemia [14].

## Current clinical translation of anti-thromboinflammatory treatment approaches

Nowadays, intravenous rt-PA and endovascular treatment (EVT) are the only treatments approved for acute ischemic stroke in Europe and the USA, although several other therapeutic approaches tackling thromboinflammation are currently under clinical evaluation.

### Anticoagulants and antithrombotics

Pharmacological agents targeting different key players along the coagulation cascade have already been well-established in the daily clinical setting to further prevent thrombosis and hemostatic disorders. However, although they are primarily used within the early phases of stroke treatment, their effect on stroke recovery and survival remains widely discussed.

#### Heparins

Through binding antithrombin-III and increasing its anticoagulative effect, unfractionated heparin and novel derivatives such as low-molecular-weight heparins (LMWH) are broadly used in different clinical scenarios. Heparins are able to exert pleiotropic anti-inflammatory effects on the immune system, e.g., by (i) interacting with various complement factors, chemokines, and cytokines, (ii) reducing inflammatory damage to the cellular glycocalyx membrane, or (iii) interfering with PAR1 dependent signaling [197]. However, administering unfractionated heparin as a periprocedural treatment to standard EVT led to no overall benefit on functional outcome 90 days post-stroke assessed by the modified Rankin Scale. Indeed, moderate-dose heparin treatment resulted in an increased ratio of intracranial hemorrhage (ISRCTN76741621) [198]. However, low-dose administration of unfractionated heparin may be beneficial periprocedurally. The previously mentioned study seems to have been analyzed in a combined manner for both moderate and low doses of unfractionated heparin and importantly, most adverse effects were only detected in the moderate dose group leading, of course, to early trial termination. Concerning LMWH, a recent meta-analysis concluded that its effect was not associated with a reduced risk of suffering a recurrent event, unfortunately together with an increase in all-cause mortality in patients older than 70 years. [199]. Up to now, long-term heparin treatment post-stroke remains not an ideal therapeutic option due to potential side-effects.

#### GPIIb/IIIa inhibitors

Tirofiban, Eptifibatide, and Abciximab are currently the most broadly extended GPIIb/IIIa inhibitors [200]. Apart from inhibiting platelet aggregation and subsequently interfering with thrombin formation, blockade of GPIIb/IIIa also leads to a reduction in IL-1β and CD40L signaling [201]. However, the potential therapeutic application of these drugs is still controversial and undergoing an intense debate. Indeed, a previous meta-analysis concluded that neither Tirofiban nor Eptifibatide led to significant long-term effects on patient outcome, pointing out an increase in mortality rate potentially caused by a higher risk of intracranial hemorrhage [202]. Contrary, further meta-analyses showed that therapeutic administration of a GPIIb/IIIa inhibitor, especially Tirofiban, was not able to increase functional outcomes 90 days after the ischemic event, although associated mortality rates were significantly decreased [200, 203, 204]. Overall, the rates of bleeding complications in the aforementioned studies remained tolerable with the exception of Abciximab, which increased the likelihood of symptomatic intracranial hemorrhage [200, 203, 204]. Therefore, recent findings propose GPIIb/IIIa-inhibitors, especially Tirofiban, as a promising therapeutic option for future clinical application in stroke recovery although larger studies are still required.

#### Thrombin and thrombin-receptor

Coagulation factor II (thrombin) regulates a vast amount of physiological effects including hemostatic balance of pro- and anti-coagulative factors, cell protection, and proinflammatory cytokine release. However, thrombin is also involved in several pathological effects primarily focused on BBB disruption, neuroinflammation, and neurotoxicity upon stroke [205]. Argatroban, more commonly used to treat heparin-induced thrombocytopenia, is a potent thrombin inhibitor. Indeed, patients treated with Argatroban showed a significant improvement in neurological outcomes without an associated increase in bleeding complications [206]. Therefore, its potential effect on long-term stroke recovery was recently under clinical evaluation although previously described improvements to functional outcomes have not been observed. This could however be attributed to high drop-out rates, a study population consisting of patients with relatively mild clinical neurological deficits (median NIHSS 8-9), and fewer patients with large vessel occlusions (NCT03740958) [207]. Similarly, acting as a thrombin receptor-inhibitor selective for PAR1, Vorapaxar showed a promising therapeutic profile when combined with acetylsalicylic-acid for a total of 60 days in patients with prior ischemic stroke [208]. However, a more comprehensive study focusing on Vorapaxar as long-term secondary stroke prevention revealed that beneficial results were limited to patients with a history of myocardial infarction (NCT00526474) [209]. Nevertheless, modulating thrombin and especially its PAR1-mediated neuroinflammatory response post-stroke should still be explored further in future studies.

#### Factor XI

Modulation of the coagulation contact pathway through FXI revealed substantial evidence of its potential therapeutic effect aiming to offer an anticoagulatory effect without affecting the extrinsic coagulation pathway, and therefore potentially minimizing the risk of severe bleeding complications [210]. In fact, deficiency in FXI has previously been associated with a decrease in overall cardiovascular events, while patients with excessive amounts of FXI presented an increased risk of ischemic stroke [210–212]. Proteomic analysis of FXIs involvement in venous thromboembolisms revealed that FXI could potentially increase inflammatory cytokine release, recruitment of leukocytes, and degranulation of neutrophils via a multitude of inflammatory signaling pathways [213]. However, patients treated with the oral FXIa inhibitor Asundexian (BAY 2433334) showed no benefit compared to non-treated patients within the scope of a randomized, placebo-controlled phase IIb trial (NCT04304508) [214]. Therefore, this therapeutic approach has recently evolved into a stroke prevention strategy rather than an acute treatment. Specifically, there is currently an active (not recruiting) phase II trial where Abelacimab (MAA868), a human monoclonal antibody selectively binding and inhibiting FXI and FXIa, is used for patients suffering from atrial fibrillation (AF) and high-risk stroke (NCT04755283) [215]. In parallel, another phase III study is also underway assessing the efficacy of Abelacimab on the rate of ischemic stroke or systemic embolism in patients with AF unsuitable for oral anticoagulation therapy (NCT05712200). Similarly, the small molecule Milvexian (BMS-986177) has also been tested as an oral FXIa inhibitor in combination with Aspirin and Clopidogrel to identify its effect on stroke prevention, although the study results are not publicly available (NCT03766581) [216]. Altogether, FXI inhibition appears to be a promising therapeutic strategy for patients at high risk of an ischemic event or related indications.

#### Glycoprotein VI (GPVI)

As previously mentioned, GPVI not only strongly contributes to thrombosis, but also to the recruitment of immune cells, increasing inflammatory signaling following an ischemic event and therefore represents a potential novel anti-thrombotic therapy that is currently under clinical assessment [44]. Glenzocimab (ACT017), a Fab fragment of humanized anti-GPVI monoclonal antibody, has been developed recently to specifically inhibit GPVI being currently under evaluation in several clinical trials [217]. In 2019, a phase II trial started where Glenzocimab was administered to stroke patients in combination with the standard-of-care, i.e., rt-PA or thrombectomy, to assess its safety profile (NCT03803007) [218]. After completion, two follow-up phase II/III trials, i.e., GREEN and ACTISAVE, are ongoing assessing the efficacy of Glenzocimab in addition to EVT aiming to be completed within 2025–2026 (NCT05559398, NCT05070260). Thus, early inhibition of GPVI appears to be one of the most promising therapeutic approaches nowadays which might be translated to clinical practice within the upcoming years.

#### Kallikrein/kinin system

As a key thromboinflammatory player, the KKS aims to be a promising therapeutic target, yet under investigation. Human tissue kallikrein (KLK)-1 activity leads to the release of bradykinin which subsequently binds to its receptor (B1R/B2R). As previously described, KLK1 triggers several signaling cascades, facilitating not only vasodilation but also anti-inflammatory effects [219]. DM-199, a recombinant form of KLK1, entered a phase II clinical trial in 2017 where its safety and tolerability were assessed in acute ischemic stroke patients (NCT03290560) [219]. Unfortunately, the results of the study remain confidential [219]. However, a follow-up phase II/III study is currently active and recruiting patients evaluating the effects of DM-199 on functional outcome, and the associated incidence of recurrent stroke in patients ineligible to receive either endovascular or thrombolysis therapy (NCT05065216). Hence, pharmacological modulation of the KKS remains, together with GPVI, as one of the most promising approaches tackling thromboinflammation.

### Anti-inflammatory approaches

As inflammatory pathways are closely connected to stroke pathogenesis, multiple anti-inflammatory strategies are currently under evaluation, focusing on either novel or repurposed drugs. Toll-like receptor (TLR)-4 is a transmembrane protein which upon activation leads to cytokine production and innate immune system response. Therefore, the novel TLR-4 antagonist ApTOLL represents a promising novel candidate for anti-inflammatory stroke therapy [220]. After a phase I clinical trial (NCT04742062) [221], ApTOLL just recently completed a more extensive clinical assessment in a phase Ib/IIa clinical study (NCT04734548) [222]. Preliminary results of this study point towards a decrease in mortality rates as well as improved functional outcomes, although final results are yet to be published [223]. Additionally, ApTOLL is currently undergoing further pre- and also clinical evaluation for its application for treating COVID-19, intracerebral hemorrhage, myocardial infarction, and multiple sclerosis.

Extensive research already exists on the role of the pro-inflammatory cytokine IL-1β in atherothrombosis and ischemic stroke. The CANTOS study tested the efficacy of Canakinumab, a monoclonal IL-1β antibody, in reducing rates of recurrent cardiovascular events and stroke among patients who already suffered a heart attack and display increased levels of high-sensitivity C-reactive protein (hsCRP) (NCT01327846) [224, 225]. Here, over 4 years, an overall reduction in cardiovascular endpoints and stroke was evident [225]. Additionally, patients treated with Canakinumab showed a dose-dependent reduction in hsCRP and IL-6 levels, while no significant change was detected in IL-18 [225, 226]. Therefore, modulation of cytokines might be a potential approach although still in a preliminary evaluation stage.

Vinpocetine has been established as a therapeutic agent modulating inflammation by inhibiting the NF-κB pathway and improving perfusion [227, 228]. Indeed, Vinpocetine-treated patients presented (i) decreased microglial and systemic inflammatory response, (ii) reduced secondary lesion growth at 7 days, and (iii) improved recovery at 90 days post-stroke, suggesting NF-κB modulation as an effective approach for stroke treatment (NCT02878772) [228]. Similarly, fingolimod, a sphingosine-1-phosphate (S1P)-receptor modulator used in the treatment of multiple sclerosis, has been tested in several clinical trials focusing on thromboinflammatory parameters. Recent data associates fingolimod administration with reduced infarct growth and improved neurological outcomes in clinical settings [13, 229]. Despite still being in an early developmental stage, anti-inflammatory treatments for long-term stroke recovery may offer a promising therapeutic strategy and should therefore be seriously considered in future clinical routine (Table 1).

**Table 1:** Overview of recent trials on anti-thromboinflammatory strategies

Name	NCT/ISRCTN	Design	Indication	Drug	Target	Phase	Status	Outcome
MR CLEAN-MED	ISRCTN76741621 [198]	Interventional, randomized, open label, blinded endpoint	Acute ischemic stroke	EVT +/- UFH +/- ASA	AT III; COX-1/-2	III	Completed /Halted	No benefit to mRS; increase in sICH
ARAIS	NCT03740958 [207]	Interventional, prospective randomized, open label, blinded endpoint	Acute ischemic stroke	Argatroban +/- rt-PA	Thrombin	IV	Completed	No benefit to mRS
PACIFIC-STROKE	NCT04304508 [214]	Interventional, randomized, quadruple-blinded, placebo-controlled	Acute non- cardioembolic ischemic stroke	Asundexian (BAY 2433334) +/- antiplatelet therapy	FXIa	II	Completed	No overall benefit
AZALEA-TIMI 71	NCT04755283 [215]	Interventional, randomized, triple-blinded	Atrial Fibrillation; Stroke	Abelacimab (MAA868) or Rivaroxaban	FXI or FX	II	Active (not recruiting)	n.a.
LILAC	NCT05712200 [215]	Interventional, randomized, double-blinded, placebo-controlled	Atrial Fibrillation	Abelacimab (MAA868)	FXI/FXIa	III	Recruiting	n.a.
AXIOMATIC-SSP	NCT03766581 [216]	Interventional, randomized, quadruple-blinded, placebo-controlled	Acute ischemic stroke; transient ischemic attack	Milvexian (BMS-986177) + Clopidogrel + ASA	FXIa	II	Completed	Results pending
ACTIMIS	NCT03803007 [217]	Interventional, randomized, triple-blinded, placebo-controlled	Acute ischemic stroke	Glenzocimab (ACT017) + rt-PA + / - Thrombectomy	GPVI	Ib/IIa	Completed	Safety confirmed
GREEN	NCT05559398 [217]	Interventional, randomized, quadruple-blinded, placebo-controlled	Acute Stroke; Ischemic Stroke	Glenzocimab (ACT017) + EVT	GPVI	II/III	Not yet recruiting	n.a.
ACTISAVE	NCT05070260 [217]	Interventional, triple-blinded, randomized, placebo-controlled	Acute ischemic stroke	Glenzocimab (ACT017) + rt-PA + / - Thrombectomy	GPVI	II/III	Recruiting	n.a.
ReMEDy1	NCT03290560 [219]	Interventional, randomized, double-blinded, placebo-controlled	Acute ischemic stroke	DM199	KKS	II	Completed	Results pending
ReMEDy2	NCT05065216	Interventional, randomized, double-blinded, placebo-controlled	Acute Stroke; Ischemic Stroke; Stroke	DM199	KKS	II/III	Active (not recruiting)	n.a.
	NCT04742062 [221]	Interventional, randomized, triple-blinded, placebo-controlled	Stroke	ApTOLL	TLR4	I	Completed	Safety confirmed
	NCT04734548 [220]	Interventional, prospective, randomized, quadruple-blinded, placebo-controlled	Stroke; acute stroke; ischemic stroke	ApTOLL + EVT (+/- rt-PA)	TLR4	Ib/IIa	Completed	Final results pending
CANTOS	NCT01327846 [225]	Interventional, randomized, triple-blinded, placebo-controlled	Atherosclerosis	Canakinumab	IL-1β	III	Completed	Reduction of cardiovascular events and hsCRP

Abbreviations: ACTIMIS Acute Ischemic Stroke Interventional Study, ACTISAVE Adaptive Efficacy and Safety Study of Glenzocimab Used as an add-on Therapy on Top of Standard of Care in the 4.5 Hours Following an Acute Ischemic Stroke, ARAIS Argatroban Plus rt-PA for Acute Ischemic Stroke, ASA acetylsalicylic acid, AT antithrombin, AZALEA-TIMI 71 Safety and Tolerability of Abelacimab (MAA868) vs. Rivaroxaban in Patients With Atrial Fibrillation, CANTOS Cardiovascular Risk Reduction Study (Reduction in Recurrent Major CV Disease Events), COX cyclooxygenase, EVT endovascular treatment, F(a) (activated) coagulation factor, GP glycoprotein, GREEN Glenzocimab for REperfusion in the Setting of Endovascular Therapy for Brain infarctioN, hsCRP high-sensitivity C-reactive protein, IL interleukin, ISRCTN international standard randomised controlled trial number (see: https://www.isrctn.com/), KKS kallikrein/kinin system, LILAC Study to evaLuate the effIcacy and Safety of abeLacimab in High-risk Patients With Atrial Fibrillation Who Have Been Deemed Unsuitable for Oral antiCoagulation, A Study on BMS-986177 for the Prevention of a Stroke in Patients Receiving Aspirin and Clopidogrel, MR CLEAN-MED, Multicenter Randomized CLinical trial of Endovascular treatment for Acute ischemic stroke in the Netherlands: the effect of periprocedural MEDication: heparin, antiplatelet agents, both or neither, mRS modified Rankin Scale, NCT national clinical trial (see: https://clinicaltrials.gov/); PACIFIC-STROKE, Study to Gather Information About Proper Dosing and Safety of the Oral FXIa Inhibitor BAY 2433334 in Patients Following a Recent Non Cardioembolic Ischemic Stroke Which Occurs When a Blood Clot Has Formed Somewhere in the Human Body (But Not in the Heart) Travelled to the Brain; ReMEDy1, Evaluation to Assess Safety and Tolerability of DM199 in Subjects With Acute Ischemic Stroke, ReMEDy2 Treatment of Acute Ischemic Stroke, rt-PA recombinant tissue plasminogen activator, sICH symptomatic intracerebral hemorrhage, TLR toll-like receptor, UFH unfractionated heparin

### Future outlook

Beyond the acute phase post-stroke, thromboinflammatory processes are involved in tissue regeneration, neuronal plasticity, cognitive recovery, and microvascular angiogenesis deeply modulating the complex ischemic cascade in a highly dynamic and time-dependent manner. However, clinical trials targeting thromboinflammation over the past decade have been less successful than expected. Stroke patients are frequently considered as sharing the same underlying pathomechanism, and therefore heterogeneity of stroke lesions has indeed been hampering the progress of clinical trials in the field [230]. Therefore, mechanism-based biomarkers which allow endophenotype patients based on their underlying pathomechanism are of urgent need. Mechanistic clustering of patients will thus lead to a homogeneous clinical trial design, reduced numbers needed to treat, and subsequently personalized therapy [231].

Novel therapeutic options within the thromboinflammatory field are currently emerging. Reduction in circulating lymphocytes using the immunosuppressant fingolimod is known to reduce infarct volume and improve neurological outcomes [13, 229]. Additionally, early inhibition of GPVI (Glenzocimab) appears to be one of the most promising approaches currently under clinical evaluation aiming to modulate the thrombotic and pro-inflammatory pathways after the ischemic event. Additionally, the novel TLR-4 antagonist ApTOLL also represents one of the first-in-line therapeutic options under development at the moment, reducing mortality rates in stroke patients. Contrary, despite neuroprotective compounds having unfortunately not yet reached clinical settings, preserving the ischemic tissue after stroke remains a solid approach. Thus, early and extremely safe interventions administered during the hyper-acute phase, even before the patient reaches the clinics, should definitely be considered in future clinical trial designs. Similarly, administering neuroprotectants during thrombolysis or thrombectomy procedures could maximally reduce reperfusion injury upon recanalization.

Importantly, in complex indications such as brain ischemia, many pathophysiologically relevant signaling mechanisms are simultaneously dysregulated. Therefore, therapeutic strategies focused on a multi-target, multi-drug approach could ultimately reverse this scenario to physiological conditions. Thus, following a multi-drug network pharmacology strategy using different drugs acting on the same causal underlying mechanism aims to be the direction for treating complex indications such as brain ischemia [232]. Overall, exploring the critical interface between neuroinflammation and thrombosis is leading to (i) a deep understanding of the underlying pathomechanism, (ii) novel therapeutic approaches, and (ii) late-stage clinical trials which will hopefully reach clinical routine and patient benefit within the upcoming years.

## References

[1] de Los Ríos la Rosa F, Khoury J, Kissela BM (2012). Eligibility for Intravenous Recombinant Tissue-Type Plasminogen Activator Within a Population: The Effect of the European Cooperative Acute Stroke Study (ECASS) III Trial. Stroke.

[2] Alexandrov AV, Grotta JC (2002). Arterial reocclusion in stroke patients treated with intravenous tissue plasminogen activator. Neurology.

[3] Leoo T, Lindgren A, Petersson J, von Arbin M (2008). Risk factors and treatment at recurrent stroke onset: results from the Recurrent Stroke Quality and Epidemiology (RESQUE) Study. Cerebrovasc Dis.

[4] Han TS, Gulli G, Fry CH (2022). Adverse consequences of immediate thrombolysis-related complications: a multi-centre registry-based cohort study of acute stroke. J Thromb Thrombolysis.

[5] Wardlaw JM, Murray V, Berge E (2012). Recombinant tissue plasminogen activator for acute ischaemic stroke: an updated systematic review and meta-analysis. Lancet.

[6] Dhanesha N, Patel RB, Doddapattar P (2022). PKM2 promotes neutrophil activation and cerebral thromboinflammation: therapeutic implications for ischemic stroke. Blood.

[7] Yao Y-Y, Wei Z-J, Zhang Y-C (2021). Functional Disability After Ischemic Stroke: A Community-Based Cross-Sectional Study in Shanghai China. Front Neurol.

[8] del Ser T, Barba R, Morin MM (2005). Evolution of cognitive impairment after stroke and risk factors for delayed progression. Stroke.

[9] Crichton SL, Bray BD, McKevitt C (2016). Patient outcomes up to 15 years after stroke: survival, disability, quality of life, cognition and mental health. J Neurol Neurosurg Psychiatry.

[10] Sun J-H, Tan L, Yu J-T (2014). Post-stroke cognitive impairment: epidemiology, mechanisms and management. Ann Transl Med.

[11] Luengo-Fernandez R, Violato M, Candio P, Leal J (2020). Economic burden of stroke across Europe: A population-based cost analysis. Eur Stroke J.

[12] Steubing RD, Szepanowski F, David C (2022). Platelet depletion does not alter long-term functional outcome after cerebral ischaemia in mice. Brain, Behavior, & Immunity - Health.

[13] Meyer SFD, De Meyer SF, Langhauser F (2022). Thromboinflammation in Brain Ischemia: Recent Updates and Future Perspectives. Stroke.

[14] Schuhmann MK, Stoll G, Bieber M (2020). CD84 Links T Cell and Platelet Activity in Cerebral Thrombo-Inflammation in Acute Stroke. Circ Res.

[15] Packham IM, Watson SP, Bicknell R, Egginton S (2014). In vivo evidence for platelet-induced physiological angiogenesis by a COX driven mechanism. PLoS One.

[16] Nording H, Baron L, Haberthür D et al (2021) The C5a/C5a receptor 1 axis controls tissue neovascularization through CXCL4 release from platelets. Nat Commun 12(1):3352. 10.1038/s41467-021-23499-w 10.1038/s41467-021-23499-w PMC8185003 34099640

[17] Planas AM (2018). Role of Immune Cells Migrating to the Ischemic Brain. Stroke.

[18] De Meyer SF, Denorme F, Langhauser F (2016). Thromboinflammation in Stroke Brain Damage. Stroke.

[19] Cowled P, Fitridge R, Thompson M (2011) Pathophysiology of reperfusion injury. In: Mechanisms of Vascular Disease: A Reference Book for Vascular Specialists [Internet]. Adelaide (AU): University of Adelaide Press 30484990

[20] Cochrane CG, Revak SD, Wuepper KD (1973). Activation of Hageman factor in solid and fluid phases. A critical role of kallikrein. J Exp Med.

[21] Austinat M, Braeuninger S, Pesquero JB (2009). Blockade of Bradykinin Receptor B1 but Not Bradykinin Receptor B2 Provides Protection From Cerebral Infarction and Brain Edema. Stroke.

[22] Göb E, Reymann S, Langhauser F (2015). Blocking of plasma kallikrein ameliorates stroke by reducing thromboinflammation. Ann Neurol.

[23] Langhauser F, Göb E, Kraft P (2012). Kininogen deficiency protects from ischemic neurodegeneration in mice by reducing thrombosis, blood-brain barrier damage, and inflammation. Blood.

[24] Albert-Weißenberger C, Sirén A-L, Kleinschnitz C (2013). Ischemic stroke and traumatic brain injury: The role of the kallikrein–kinin system. Prog Neurobiol.

[25] Ottaiano TF, Andrade SS, de Oliveira C (2017). Plasma kallikrein enhances platelet aggregation response by subthreshold doses of ADP. Biochimie.

[26] Marcos-Contreras OA, Martinez de Lizarrondo S, Bardou I (2016). Hyperfibrinolysis increases blood-brain barrier permeability by a plasmin- and bradykinin-dependent mechanism. Blood.

[27] Simão F, Ustunkaya T, Clermont AC, Feener EP (2017). Plasma kallikrein mediates brain hemorrhage and edema caused by tissue plasminogen activator therapy in mice after stroke. Blood.

[28] Ansari J, Gavins FNE (2021). The impact of thrombo-inflammation on the cerebral microcirculation. Microcirculation.

[29] Jackson SP, Darbousset R, Schoenwaelder SM (2019). Thromboinflammation: challenges of therapeutically targeting coagulation and other host defense mechanisms. Blood.

[30] Hoque MM, Abdelazim H, Jenkins-Houk C (2021). The cerebral microvasculature: Basic and clinical perspectives on stroke and glioma. Microcirculation.

[31] Gavins F, Yilmaz G, Granger DN (2007). The evolving paradigm for blood cell-endothelial cell interactions in the cerebral microcirculation. Microcirculation.

[32] Umemura A, Yamada K, Mabe H, Nagai H (1997). Production of platelet-activating factor during focal cerebral ischemia and reperfusion in the rat. J Stroke Cerebrovasc Dis.

[33] EC de B T, de Brito Toscano EC, Silva BC (2016). Platelet-activating factor receptor (PAFR) plays a crucial role in experimental global cerebral ischemia and reperfusion. Brain Res Bull.

[34] Hayon Y, Dashevsky O, Shai E (2012). Platelet microparticles induce angiogenesis and neurogenesis after cerebral ischemia. Curr Neurovasc Res.

[35] Hayon Y, Dashevsky O, Shai E (2013). Platelet lysates stimulate angiogenesis, neurogenesis and neuroprotection after stroke. Thromb Haemost.

[36] Kocovski P, Jiang X, D’Souza C (2019). Platelet Depletion is Effective in Ameliorating Anxiety-Like Behavior and Reducing the Pro-Inflammatory Environment in the Hippocampus in Murine Experimental Autoimmune Encephalomyelitis. J Clin Med.

[37] Schafer AI (2001). Thrombocytosis and thrombocythemia. Blood Rev.

[38] Kanaji S, Fahs SA, Shi Q (2012). Contribution of platelet vs. endothelial VWF to platelet adhesion and hemostasis. J Thromb Haemost.

[39] Savage B, Saldívar E, Ruggeri ZM (1996). Initiation of platelet adhesion by arrest onto fibrinogen or translocation on von Willebrand factor. Cell.

[40] Berndt MC, Shen Y, Dopheide SM (2001). The vascular biology of the glycoprotein Ib-IX-V complex. Thromb Haemost.

[41] Kleinschnitz C, De Meyer SF, Schwarz T (2009). Deficiency of von Willebrand factor protects mice from ischemic stroke. Blood.

[42] Massberg S, Gawaz M, Grüner S (2003). A Crucial Role of Glycoprotein VI for Platelet Recruitment to the Injured Arterial Wall In Vivo. J Exp Med.

[43] Schuhmann MK, Guthmann J, Stoll G (2017). Blocking of platelet glycoprotein receptor Ib reduces “thrombo-inflammation” in mice with acute ischemic stroke. J Neuroinflammation.

[44] Rayes J, Watson SP, Nieswandt B (2019). Functional significance of the platelet immune receptors GPVI and CLEC-2. J Clin Invest.

[45] Mammadova-Bach E, Ollivier V, Loyau S (2015). Platelet glycoprotein VI binds to polymerized fibrin and promotes thrombin generation. Blood.

[46] Alshehri OM, Hughes CE, Montague S (2015). Fibrin activates GPVI in human and mouse platelets. Blood.

[47] Braun A, Vogtle T, Varga-Szabo D, Nieswandt B (2011). STIM and Orai in hemostasis and thrombosis. Front Biosci.

[48] Bergmeier W, Stefanini L (2013). Platelet ITAM signaling. Curr Opin Hematol.

[49] Nieswandt B, Watson SP (2003). Platelet-collagen interaction: is GPVI the central receptor?. Blood.

[50] Dütting S, Bender M, Nieswandt B (2012). Platelet GPVI: a target for antithrombotic therapy?!. Trends Pharmacol Sci.

[51] Müller F, Mutch NJ, Schenk WA (2009). Platelet polyphosphates are proinflammatory and procoagulant mediators in vivo. Cell.

[52] Goebel S, Li Z, Vogelmann J (2013). The GPVI-Fc fusion protein Revacept improves cerebral infarct volume and functional outcome in stroke. PLoS One.

[53] Kleinschnitz C, Pozgajova M, Pham M (2007). Targeting platelets in acute experimental stroke: impact of glycoprotein Ib, VI, and IIb/IIIa blockade on infarct size, functional outcome, and intracranial bleeding. Circulation.

[54] Heydenreich N, Nolte MW, Göb E (2012). C1-inhibitor protects from brain ischemia-reperfusion injury by combined antiinflammatory and antithrombotic mechanisms. Stroke.

[55] Shattil SJ, Kim C, Ginsberg MH (2010). The final steps of integrin activation: the end game. Nat Rev Mol Cell Biol.

[56] Kraft P, Schuhmann MK, Fluri F (2015). Efficacy and Safety of Platelet Glycoprotein Receptor Blockade in Aged and Comorbid Mice With Acute Experimental Stroke. Stroke.

[57] Adams HP, Effron MB, Torner J (2008). Emergency administration of abciximab for treatment of patients with acute ischemic stroke: results of an international phase III trial: Abciximab in Emergency Treatment of Stroke Trial (AbESTT-II). Stroke.

[58] Kellert L, Hametner C, Rohde S (2013). Endovascular stroke therapy: tirofiban is associated with risk of fatal intracerebral hemorrhage and poor outcome. Stroke.

[59] Ma Y, Liu Y, Zhang Z, Yang G-Y (2019). Significance of Complement System in Ischemic Stroke: A Comprehensive Review. Aging Dis.

[60] Komotar RJ, Kim GH, Otten ML (2008). The role of complement in stroke therapy. Adv Exp Med Biol.

[61] Martel C, Cointe S, Maurice P (2011). Requirements for membrane attack complex formation and anaphylatoxins binding to collagen-activated platelets. PLoS One.

[62] Ritis K, Doumas M, Mastellos D (2006). A novel C5a receptor-tissue factor cross-talk in neutrophils links innate immunity to coagulation pathways. J Immunol.

[63] Wojta J, Kaun C, Zorn G (2002). C5a stimulates production of plasminogen activator inhibitor-1 in human mast cells and basophils. Blood.

[64] Kastl SP, Speidl WS, Kaun C, Rega G, Assadian A, Weiss TW, Valent P, Hagmueller GW, Maurer G, Huber K, Wojta J (2006). The complement component C5a induces the expression of plasminogen activator inhibitor-1 in human macrophages via NF-κB activation. J Thromb Haemost.

[65] Sims PJ, Wiedmer T (1991). The response of human platelets to activated components of the complement system. Immunol Today.

[66] Hamilton KK, Hattori R, Esmon CT, Sims PJ (1990). Complement proteins C5b-9 induce vesiculation of the endothelial plasma membrane and expose catalytic surface for assembly of the prothrombinase enzyme complex. J Biol Chem.

[67] Oikonomopoulou K, Ricklin D, Ward PA, Lambris JD (2012). Interactions between coagulation and complement--their role in inflammation. Semin Immunopathol.

[68] Krisinger MJ, Goebeler V, Lu Z (2012). Thrombin generates previously unidentified C5 products that support the terminal complement activation pathway. Blood.

[69] Cowell RM, Plane JM, Silverstein FS (2003). Complement activation contributes to hypoxic-ischemic brain injury in neonatal rats. J Neurosci.

[70] Komotar RJ, Starke RM, Arias EJ (2009). The complement cascade: new avenues in stroke therapy. Curr Vasc Pharmacol.

[71] Arumugam TV, Woodruff TM, Lathia JD (2009). Neuroprotection in stroke by complement inhibition and immunoglobulin therapy. Neuroscience.

[72] Thom V, Arumugam TV, Magnus T, Gelderblom M (2017). Therapeutic Potential of Intravenous Immunoglobulin in Acute Brain Injury. Front Immunol.

[73] Stokowska A, Olsson S, Holmegaard L (2011). Plasma C3 and C3a levels in cryptogenic and large-vessel disease stroke: associations with outcome. Cerebrovasc Dis.

[74] Pedersen ED, Løberg EM, Vege E (2009). In situ deposition of complement in human acute brain ischaemia. Scand J Immunol.

[75] Ho-Tin-Noé B, Jadoui S (2018). Spontaneous bleeding in thrombocytopenia: Is it really spontaneous?. Transfus Clin Biol.

[76] Langer HF, Gawaz M (2008). Platelet-vessel wall interactions in atherosclerotic disease. Thromb Haemost.

[77] Braun LJ, Stegmeyer RI, Schäfer K (2020). Platelets docking to VWF prevent leaks during leukocyte extravasation by stimulating Tie-2. Blood.

[78] Gros A, Syvannarath V, Lamrani L (2015). Single platelets seal neutrophil-induced vascular breaches via GPVI during immune-complex-mediated inflammation in mice. Blood.

[79] Said S, Rosenblum WI, Povlishock JT, Nelson GH (1993). Correlations between morphological changes in platelet aggregates and underlying endothelial damage in cerebral microcirculation of mice. Stroke.

[80] Rosenblum WI (1997). Platelet adhesion and aggregation without endothelial denudation or exposure of basal lamina and/or collagen. J Vasc Res.

[81] Tang YH, Vital S, Russell J (2014). Transient ischemia elicits a sustained enhancement of thrombus development in the cerebral microvasculature: effects of anti-thrombotic therapy. Exp Neurol.

[82] Leiter O, Seidemann S, Overall RW (2019). Exercise-Induced Activated Platelets Increase Adult Hippocampal Precursor Proliferation and Promote Neuronal Differentiation. Stem Cell Reports.

[83] Mazzucco L, Borzini P, Gope R (2010). Platelet-Derived Factors Involved in Tissue Repair—From Signal to Function. Transfus Med Rev.

[84] Sun P, Zhang K, Hassan SH (2020). Endothelium-Targeted Deletion of microRNA-15a/16-1 Promotes Poststroke Angiogenesis and Improves Long-Term Neurological Recovery. Circ Res.

[85] Brill A (2004). Differential role of platelet granular mediators in angiogenesis. Cardiovasc Res.

[86] Li L, Jiang Q, Zhang L (2007). Angiogenesis and improved cerebral blood flow in the ischemic boundary area detected by MRI after administration of sildenafil to rats with embolic stroke. Brain Res.

[87] Jickling GC, Liu D, Ander BP (2015). Targeting neutrophils in ischemic stroke: translational insights from experimental studies. J Cereb Blood Flow Metab.

[88] Xu X, Jiang Y (2014). The Yin and Yang of innate immunity in stroke. Biomed Res Int.

[89] Sadik CD, Kim ND, Luster AD (2011). Neutrophils cascading their way to inflammation. Trends Immunol.

[90] Reichel CA, Khandoga A, Anders H-J (2006). Chemokine receptors Ccr1, Ccr2, and Ccr5 mediate neutrophil migration to postischemic tissue. J Leukoc Biol.

[91] Dimitrijevic OB, Stamatovic SM, Keep RF, Andjelkovic AV (2007). Absence of the chemokine receptor CCR2 protects against cerebral ischemia/reperfusion injury in mice. Stroke.

[92] Zhang RL, Chopp M, Jiang N (1995). Anti-intercellular adhesion molecule-1 antibody reduces ischemic cell damage after transient but not permanent middle cerebral artery occlusion in the Wistar rat. Stroke.

[93] Jiang N, Moyle M, Soule HR (1995). Neutrophil inhibitory factor is neuroprotective after focal ischemia in rats. Ann Neurol.

[94] Huang J, Choudhri TF, Winfree CJ (2000). Postischemic cerebrovascular E-selectin expression mediates tissue injury in murine stroke. Stroke.

[95] Furuya K, Takeda H, Azhar S (2001). Examination of several potential mechanisms for the negative outcome in a clinical stroke trial of enlimomab, a murine anti-human intercellular adhesion molecule-1 antibody: a bedside-to-bench study. Stroke.

[96] Becker KJ (2002). Anti-leukocyte antibodies: LeukArrest (Hu23F2G) and Enlimomab (R6.5) in acute stroke. Curr Med Res Opin.

[97] Bednar MM, Gross CE, Russell SR (1998). Humanized anti-L-selectin monoclonal antibody DREG200 therapy in acute thromboembolic stroke. Neurol Res.

[98] Neumann J, Riek-Burchardt M, Herz J (2015). Very-late-antigen-4 (VLA-4)-mediated brain invasion by neutrophils leads to interactions with microglia, increased ischemic injury and impaired behavior in experimental stroke. Acta Neuropathol.

[99] Kang L, Yu H, Yang X (2020). Neutrophil extracellular traps released by neutrophils impair revascularization and vascular remodeling after stroke. Nat Commun.

[100] Erdener ŞE, Tang J, Kılıç K (2021). Dynamic capillary stalls in reperfused ischemic penumbra contribute to injury: A hyperacute role for neutrophils in persistent traffic jams. J Cereb Blood Flow Metab.

[101] Zrzavy T, Machado-Santos J, Christine S (2018). Dominant role of microglial and macrophage innate immune responses in human ischemic infarcts. Brain Pathol.

[102] Otxoa-de-Amezaga A, Miró-Mur F, Pedragosa J (2019). Microglial cell loss after ischemic stroke favors brain neutrophil accumulation. Acta Neuropathol.

[103] Perez-de-Puig I, Miró-Mur F, Ferrer-Ferrer M (2015). Neutrophil recruitment to the brain in mouse and human ischemic stroke. Acta Neuropathol.

[104] Price CJS, Menon DK, Peters AM (2004). Cerebral neutrophil recruitment, histology, and outcome in acute ischemic stroke: an imaging-based study. Stroke.

[105] Semerano A, Laredo C, Zhao Y (2019). Leukocytes, Collateral Circulation, and Reperfusion in Ischemic Stroke Patients Treated With Mechanical Thrombectomy. Stroke.

[106] Garcia-Bonilla L, Moore JM, Racchumi G (2014). Inducible nitric oxide synthase in neutrophils and endothelium contributes to ischemic brain injury in mice. J Immunol.

[107] Massberg S, Grahl L, von Bruehl M-L (2010). Reciprocal coupling of coagulation and innate immunity via neutrophil serine proteases. Nat Med.

[108] von Brühl M-L, Stark K, Steinhart A (2012). Monocytes, neutrophils, and platelets cooperate to initiate and propagate venous thrombosis in mice in vivo. J Exp Med.

[109] Kim S-W, Lee H, Lee H-K (2019). Neutrophil extracellular trap induced by HMGB1 exacerbates damages in the ischemic brain. Acta Neuropathol Commun.

[110] Martinod K, Wagner DD (2014). Thrombosis: tangled up in NETs. Blood.

[111] Enzmann G, Mysiorek C, Gorina R (2013). The neurovascular unit as a selective barrier to polymorphonuclear granulocyte (PMN) infiltration into the brain after ischemic injury. Acta Neuropathol.

[112] ElAli A, LeBlanc NJ (2016). The Role of Monocytes in Ischemic Stroke Pathobiology: New Avenues to Explore. Frontiers in Aging. Neuroscience.

[113] Wattananit S, Tornero D, Graubardt N (2016). Monocyte-Derived Macrophages Contribute to Spontaneous Long-Term Functional Recovery after Stroke in Mice. J Neurosci.

[114] Werner Y, Mass E, Ashok Kumar P (2020). Cxcr4 distinguishes HSC-derived monocytes from microglia and reveals monocyte immune responses to experimental stroke. Nat Neurosci.

[115] Che X, Ye W, Panga L (2001). Monocyte chemoattractant protein-1 expressed in neurons and astrocytes during focal ischemia in mice. Brain Research.

[116] Chu HX, Arumugam TV, Gelderblom M (2014). Role of CCR2 in inflammatory conditions of the central nervous system. J Cereb Blood Flow Metab.

[117] Fang W, Zhai X, Han D (2018). CCR2-dependent monocytes/macrophages exacerbate acute brain injury but promote functional recovery after ischemic stroke in mice. Theranostics.

[118] Schmidt A, Strecker J-K, Hucke S (2017). Targeting Different Monocyte/Macrophage Subsets Has No Impact on Outcome in Experimental Stroke. Stroke.

[119] Ajmo CT, Vernon DOL, Collier L (2008). The spleen contributes to stroke-induced neurodegeneration. J Neurosci Res.

[120] Gliem M, Mausberg AK, Lee J-I (2012). Macrophages prevent hemorrhagic infarct transformation in murine stroke models. Ann Neurol.

[121] Chu HX, Broughton BRS, Kim HA (2015). Evidence That Ly6C(hi) Monocytes are Protective in Acute Ischemic Stroke by Promoting M2 Macrophage Polarization. Stroke.

[122] Pedragosa J, Miró-Mur F, Otxoa-de-Amezaga A (2020). CCR2 deficiency in monocytes impairs angiogenesis and functional recovery after ischemic stroke in mice. J Cereb Blood Flow Metab.

[123] Kim E, Yang J, Beltran CD, Cho S (2014). Role of spleen-derived monocytes/macrophages in acute ischemic brain injury. J Cereb Blood Flow Metab.

[124] Schilling M, Strecker J-K, Ringelstein EB (2009). The role of CC chemokine receptor 2 on microglia activation and blood-borne cell recruitment after transient focal cerebral ischemia in mice. Brain Res.

[125] Perego C, Fumagalli S, Zanier ER (2016). Macrophages are essential for maintaining a M2 protective response early after ischemic brain injury. Neurobiol Dis.

[126] Gelderblom M, Weymar A, Bernreuther C (2012). Neutralization of the IL-17 axis diminishes neutrophil invasion and protects from ischemic stroke. Blood.

[127] Shichita T, Sugiyama Y, Ooboshi H (2009). Pivotal role of cerebral interleukin-17-producing gammadeltaT cells in the delayed phase of ischemic brain injury. Nat Med.

[128] Vindegaard N, Muñoz-Briones C, El Ali HH (2017). T-cells and macrophages peak weeks after experimental stroke: Spatial and temporal characteristics. Neuropathology.

[129] Xie L, Li W, Hersh J (2019). Experimental ischemic stroke induces long-term T cell activation in the brain. J Cereb Blood Flow Metab.

[130] Morrell CN, Sun H, Swaim AM, Baldwin WM 3rd (2007) Platelets an inflammatory force in transplantation. Am J Transplant 7(11):2447–54. 10.1111/j.1600-6143.2007.01958.x 10.1111/j.1600-6143.2007.01958.x 17927608

[131] Magnus T, Wiendl H, Kleinschnitz C (2012). Immune mechanisms of stroke. Curr Opin Neurol.

[132] Kleinschnitz C, Schwab N, Kraft P (2010). Early detrimental T-cell effects in experimental cerebral ischemia are neither related to adaptive immunity nor thrombus formation. Blood.

[133] Duffy SS, Keating BA, Perera CJ, Moalem-Taylor G (2018). The role of regulatory T cells in nervous system pathologies. J Neurosci Res.

[134] Kleinschnitz C, Kraft P, Dreykluft A (2013). Regulatory T cells are strong promoters of acute ischemic stroke in mice by inducing dysfunction of the cerebral microvasculature. Blood.

[135] Schuhmann MK, Kraft P, Stoll G (2015). CD28 superagonist-mediated boost of regulatory T cells increases thrombo-inflammation and ischemic neurodegeneration during the acute phase of experimental stroke. J Cereb Blood Flow Metab.

[136] Zhang H, Xia Y, Ye Q (2018). Expansion of Regulatory T Cells with IL-2/IL-2 Antibody Complex Protects against Transient Ischemic Stroke. J Neurosci.

[137] Ren X, Akiyoshi K, Vandenbark AA (2011). CD4 FoxP3 regulatory T-cells in cerebral ischemic stroke. Metab Brain Dis.

[138] Stubbe T, Ebner F, Richter D (2013). Regulatory T cells accumulate and proliferate in the ischemic hemisphere for up to 30 days after MCAO. J Cereb Blood Flow Metab.

[139] Shi L, Sun Z, Su W (2021). Treg cell-derived osteopontin promotes microglia-mediated white matter repair after ischemic stroke. Immunity.

[140] Ito M, Komai K, Mise-Omata S (2019). Brain regulatory T cells suppress astrogliosis and potentiate neurological recovery. Nature.

[141] Hug A, Dalpke A, Wieczorek N (2009). Infarct volume is a major determiner of post-stroke immune cell function and susceptibility to infection. Stroke.

[142] Yan J, Read SJ, Henderson RD (2012). Frequency and function of regulatory T cells after ischaemic stroke in humans. J Neuroimmunol.

[143] Yan J, Greer JM, Etherington K (2009). Immune activation in the peripheral blood of patients with acute ischemic stroke. J Neuroimmunol.

[144] Pang X, Qian W (2017). Changes in Regulatory T-Cell Levels in Acute Cerebral Ischemia. J Neurol Surg A Cent Eur Neurosurg.

[145] Voskoboinik I, Whisstock JC, Trapani JA (2015). Perforin and granzymes: function, dysfunction and human pathology. Nat Rev Immunol.

[146] Liesz A, Zhou W, Mracskó É (2011). Inhibition of lymphocyte trafficking shields the brain against deleterious neuroinflammation after stroke. Brain.

[147] Mracsko E, Liesz A, Stojanovic A (2014). Antigen dependently activated cluster of differentiation 8-positive T cells cause perforin-mediated neurotoxicity in experimental stroke. J Neurosci.

[148] Selvaraj UM, Ujas TA, Kong X (2021). Delayed diapedesis of CD8 T cells contributes to long-term pathology after ischemic stroke in male mice. Brain Behav Immun.

[149] Adelson JD, Barreto GE, Xu L (2012). Neuroprotection from stroke in the absence of MHCI or PirB. Neuron.

[150] Miró-Mur F, Urra X, Ruiz-Jaén F (2020). Antigen-Dependent T Cell Response to Neural Peptides After Human Ischemic Stroke. Front Cell Neurosci.

[151] Selvaraj UM, Poinsatte K, Torres V (2016). Heterogeneity of B Cell Functions in Stroke-Related Risk, Prevention, Injury, and Repair. Neurotherapeutics.

[152] Fauchais A-L, Lalloué F, Lise M-C (2008). Role of endogenous brain-derived neurotrophic factor and sortilin in B cell survival. J Immunol.

[153] Doyle KP, Quach LN, Solé M (2015). B-lymphocyte-mediated delayed cognitive impairment following stroke. J Neurosci.

[154] Schuhmann MK, Langhauser F, Kraft P, Kleinschnitz C (2017). B cells do not have a major pathophysiologic role in acute ischemic stroke in mice. J Neuroinflammation.

[155] Chen Y, Bodhankar S, Murphy SJ (2012). Intrastriatal B-cell administration limits infarct size after stroke in B-cell deficient mice. Metab Brain Dis.

[156] Ren X, Akiyoshi K, Dziennis S (2011). Regulatory B Cells Limit CNS Inflammation and Neurologic Deficits in Murine Experimental Stroke. J Neurosci.

[157] Bodhankar S, Chen Y, Lapato A (2015). Regulatory CD8(+)CD122 (+) T-cells predominate in CNS after treatment of experimental stroke in male mice with IL-10-secreting B-cells. Metab Brain Dis.

[158] Neumann J, Henneberg S, von Kenne S (2018). Beware the intruder: Real time observation of infiltrated neutrophils and neutrophil-Microglia interaction during stroke in vivo. PLoS One.

[159] Ortega SB, Torres VO, Latchney SE (2020). B cells migrate into remote brain areas and support neurogenesis and functional recovery after focal stroke in mice. Proc Natl Acad Sci U S A.

[160] Gear ARL, Camerini D (2003). Platelet chemokines and chemokine receptors: linking hemostasis, inflammation, and host defense. Microcirculation.

[161] von Hundelshausen P, Weber KSC (2001). RANTES Deposition by Platelets Triggers Monocyte Arrest on Inflamed and Atherosclerotic Endothelium. Circulation.

[162] Schober A, Manka D, von Hundelshausen P (2002). Deposition of Platelet RANTES Triggering Monocyte Recruitment Requires P-Selectin and Is Involved in Neointima Formation After Arterial Injury. Circulation.

[163] Weyrich AS, Elstad MR, McEver RP (1996). Activated platelets signal chemokine synthesis by human monocytes. J Clin Investig.

[164] Brandt E, Petersen F, Ludwig A (2000). The β-thromboglobulins and platelet factor 4: blood platelet-derived CXC chemokines with divergent roles in early neutrophil regulation. J Leukoc Biol.

[165] Assoian RK, Sporn MB (1986). Type beta transforming growth factor in human platelets: release during platelet degranulation and action on vascular smooth muscle cells. J Cell Biol.

[166] Scheuerer B, Ernst M, Dürrbaum-Landmann I (2000). The CXC-chemokine platelet factor 4 promotes monocyte survival and induces monocyte differentiation into macrophages. Blood.

[167] Cognasse F, Hamzeh-Cognasse H, Lafarge S (2007). Human platelets can activate peripheral blood B cells and increase production of immunoglobulins. Exp Hematol.

[168] Lindemann S, Tolley ND, Dixon DA (2001). Activated platelets mediate inflammatory signaling by regulated interleukin 1beta synthesis. J Cell Biol.

[169] Albelda SM, Wayne Smith C, Ward PA (1994). Adhesion molecules and inflammatory injury. The FASEB J.

[170] May AE, Langer H, Seizer P (2007). Platelet-leukocyte interactions in inflammation and atherothrombosis. Semin Thromb Hemost.

[171] Zarbock A, Polanowska-Grabowska RK, Ley K (2007). Platelet-neutrophil-interactions: linking hemostasis and inflammation. Blood Rev.

[172] Wang Y, Sakuma M, Chen Z (2005). Leukocyte engagement of platelet glycoprotein Ibalpha via the integrin Mac-1 is critical for the biological response to vascular injury. Circulation.

[173] Issekutz AC, Rowter D, Springer TA (1999). Role of ICAM-1 and ICAM-2 and alternate CD11/CD18 ligands in neutrophil transendothelial migration. J Leukoc Biol.

[174] Schuhmann MK, Bieber M, Franke M (2021). Platelets and lymphocytes drive progressive penumbral tissue loss during middle cerebral artery occlusion in mice. J Neuroinflammation.

[175] Denorme F, Vanhoorelbeke K, De Meyer SF (2019). von Willebrand Factor and Platelet Glycoprotein Ib: A Thromboinflammatory Axis in Stroke. Front Immunol.

[176] McEver RP, Martin MN (1984). A monoclonal antibody to a membrane glycoprotein binds only to activated platelets. J Biol Chem.

[177] Berman CL, Yeo EL, Wencel-Drake JD (1986). A platelet alpha granule membrane protein that is associated with the plasma membrane after activation. Characterization and subcellular localization of platelet activation-dependent granule-external membrane protein. J Clin Investig.

[178] Lam FW, Burns AR, Smith CW, Rumbaut RE (2011). Platelets enhance neutrophil transendothelial migration via P-selectin glycoprotein ligand-1. Am J Physiol Heart Circ Physiol.

[179] Ma Y-Q, Plow EF, Geng J-G (2004). P-selectin binding to P-selectin glycoprotein ligand-1 induces an intermediate state of αMβ2 activation and acts cooperatively with extracellular stimuli to support maximal adhesion of human neutrophils. Blood.

[180] Irving PM, Macey MG, Shah U (2004). Formation of platelet-leukocyte aggregates in inflammatory bowel disease. Inflamm Bowel Dis.

[181] Rawish E, Nording H, Münte T, Langer HF (2020). Platelets as Mediators of Neuroinflammation and Thrombosis. Front Immunol.

[182] Nording H, Sauter M, Lin C (2022). Activated Platelets Upregulate β Integrin Mac-1 (CD11b/CD18) on Dendritic Cells, Which Mediates Heterotypic Cell-Cell Interaction. J Immunol.

[183] Burger PC, Wagner DD (2003). Platelet P-selectin facilitates atherosclerotic lesion development. Blood.

[184] Dong ZM, Brown AA, Wagner DD (2000). Prominent Role of P-Selectin in the Development of Advanced Atherosclerosis in ApoE-Deficient Mice. Circulation.

[185] Manka D, Collins RG, Ley K (2001). Absence of p-selectin, but not intercellular adhesion molecule-1, attenuates neointimal growth after arterial injury in apolipoprotein e-deficient mice. Circulation.

[186] Htun P, Fateh-Moghadam S, Tomandl B (2006). Course of platelet activation and platelet-leukocyte interaction in cerebrovascular ischemia. Stroke.

[187] Dixon DA (2006). Expression of COX-2 in platelet-monocyte interactions occurs via combinatorial regulation involving adhesion and cytokine signaling. J Clin Investig.

[188] FitzGerald GA (2003). COX-2 and beyond: Approaches to prostaglandin inhibition in human disease. Nat Rev Drug Discov.

[189] Schuett H, Luchtefeld M, Grothusen C (2009). How much is too much? Interleukin-6 and its signalling in atherosclerosis. Thrombosis and Haemostasis.

[190] Henn V, Slupsky JR, Gräfe M (1998). CD40 ligand on activated platelets triggers an inflammatory reaction of endothelial cells. Nature.

[191] Armitage RJ, Fanslow WC, Strockbine L (1992). Molecular and biological characterization of a murine ligand for CD40. Nature.

[192] Foy TM, Aruffo A, Bajorath J (1996). Immune regulation by CD40 and its ligand GP39. Annu Rev Immunol.

[193] Khan SY, Kelher MR, Heal JM (2006). Soluble CD40 ligand accumulates in stored blood components, primes neutrophils through CD40, and is a potential cofactor in the development of transfusion-related acute lung injury. Blood.

[194] Antoniades C, Bakogiannis C, Tousoulis D (2009). The CD40/CD40 ligand system: linking inflammation with atherothrombosis. J Am Coll Cardiol.

[195] Zhu L, Huang Z, Stålesen R (2014). Platelets provoke distinct dynamics of immune responses by differentially regulating CD4+ T-cell proliferation. J Thromb Haemost.

[196] Zhou L, Lopes JE, Chong MMW (2008). TGF-β-induced Foxp3 inhibits TH17 cell differentiation by antagonizing RORγt function. Nature.

[197] Beurskens DMH, Huckriede JP, Schrijver R (2020). The Anticoagulant and Nonanticoagulant Properties of Heparin. Thromb Haemost.

[198] van der Steen W, van de Graaf RA, Chalos V (2022). Safety and efficacy of aspirin, unfractionated heparin, both, or neither during endovascular stroke treatment (MR CLEAN-MED): an open-label, multicentre, randomised controlled trial. Lancet.

[199] Ye Y, Zhou W, Cheng W, Liu Y, Chang R (2020). Short-Term and Long-Term Safety and Efficacy of Treatment of Acute Ischemic Stroke with Low-Molecular-Weight Heparin: Meta-Analysis of 19 Randomized Controlled Trials. World Neurosurg.

[200] Zhu X, Cao G (2020). Safety of Glycoprotein IIb-IIIa Inhibitors Used in Stroke-Related Treatment: A Systematic Review and Meta-Analysis. Clin Appl Thromb Hemost.

[201] Quinn MJ, Plow EF, Topol EJ (2002). Platelet glycoprotein IIb/IIIa inhibitors: recognition of a two-edged sword?. Circulation.

[202] Liu J, Yang Y, Liu H (2022). Efficacy outcomes and safety measures of intravenous tirofiban or eptifibatide for patients with acute ischemic stroke: a systematic review and meta-analysis of prospective studies. J Thromb Thrombolysis.

[203] Zhang A, Wu N, Liu X, Jiang T (2022). Continuous intravenous tirofiban can improve the 90-day functional outcome and decrease 90-day mortality without increasing bleeding risk in acute ischemic stroke patients treated by endovascular therapy: A meta-analysis. J Clin Neurosci.

[204] Tang L, Tang X, Yang Q (2021). The Application of Tirofiban in the Endovascular Treatment of Acute Ischemic Stroke: A Meta-Analysis. Cerebrovasc Dis.

[205] Shlobin NA, Har-Even M, Itsekson-Hayosh ZE (2021). Role of Thrombin in Central Nervous System Injury and Disease. Biomolecules.

[206] Hou X, Jin C, Pan C (2021). Effects of argatroban therapy for stroke patients: A meta-analysis. J Clin Neurosci.

[207] Chen H-S, Cui Y, Zhou Z-H (2023). Effect of Argatroban Plus Intravenous Alteplase vs Intravenous Alteplase Alone on Neurologic Function in Patients With Acute Ischemic Stroke: The ARAIS Randomized Clinical Trial. JAMA.

[208] Shinohara Y, Goto S, Doi M, Jensen P (2012). Safety of the novel protease-activated receptor-1 antagonist vorapaxar in Japanese patients with a history of ischemic stroke. J Stroke Cerebrovasc Dis.

[209] Morrow DA, Braunwald E, Bonaca MP (2012). Vorapaxar in the secondary prevention of atherothrombotic events. N Engl J Med.

[210] DeLoughery EP, Olson SR, Puy C (2019). The Safety and Efficacy of Novel Agents Targeting Factors XI and XII in Early Phase Human Trials. Semin Thromb Hemost.

[211] Preis M, Hirsch J, Kotler A (2017). Factor XI deficiency is associated with lower risk for cardiovascular and venous thromboembolism events. Blood.

[212] Gill D, Georgakis MK, Laffan M (2018). Genetically Determined FXI (Factor XI) Levels and Risk of Stroke. Stroke.

[213] Pallares Robles A, Ten Cate V, Schulz A (2022). Association of FXI activity with thrombo-inflammation, extracellular matrix, lipid metabolism and apoptosis in venous thrombosis. Sci Rep.

[214] Shoamanesh A, Mundl H, Smith EE (2022). Factor XIa inhibition with asundexian after acute non-cardioembolic ischaemic stroke (PACIFIC-Stroke): an international, randomised, double-blind, placebo-controlled, phase 2b trial. Lancet.

[215] Koch AW, Schiering N, Melkko S (2019). MAA868, a novel FXI antibody with a unique binding mode, shows durable effects on markers of anticoagulation in humans. Blood.

[216] Sharma M, Molina CA, Toyoda K (2022). Rationale and design of the AXIOMATIC-SSP phase II trial: Antithrombotic treatment with factor XIa inhibition to Optimize Management of Acute Thromboembolic events for Secondary Stroke Prevention. J Stroke Cerebrovasc Dis.

[217] Wichaiyo S, Parichatikanond W, Rattanavipanon W (2022) Glenzocimab: A GPVI (Glycoprotein VI)-Targeted Potential Antiplatelet Agent for the Treatment of Acute Ischemic Stroke. Stroke 53:3506–3513 10.1161/STROKEAHA.122.039790 36128904

[218] Voors-Pette C, Lebozec K, Dogterom P (2019). Safety and Tolerability, Pharmacokinetics, and Pharmacodynamics of ACT017, an Antiplatelet GPVI (Glycoprotein VI) Fab. Arterioscler Thromb Vasc Biol.

[219] Alexander-Curtis M, Pauls R, Chao J (2019). Human tissue kallikrein in the treatment of acute ischemic stroke. Ther Adv Neurol Disord.

[220] Durán-Laforet V, Peña-Martínez C, García-Culebras A (2021). Pathophysiological and pharmacological relevance of TLR4 in peripheral immune cells after stroke. Pharmacol Ther.

[221] Hernández-Jiménez M, Martín-Vílchez S, Ochoa D (2022). First-in-human phase I clinical trial of a TLR4-binding DNA aptamer, ApTOLL: Safety and pharmacokinetics in healthy volunteers. Mol Ther Nucleic Acids.

[222] Hernández-Jiménez M, Abad-Santos F, Cotgreave I (2023). APRIL: A double-blind, placebo-controlled, randomized, Phase Ib/IIa clinical study of ApTOLL for the treatment of acute ischemic stroke. Front Neurol.

[223] (2023) ApTOLL reduces mortality from 18% to 5% in a Phase 1b/2a trial in ischemic stroke patients. In: Aptatargets. https://aptatargets.com/newsroom/aptoll-reduces-mortality-from-18-to-5-in-a-phase-1b-2a-trial-in-ischemic-stroke-patients/. Accessed 12 Apr 2023

[224] Ridker PM, Thuren T, Zalewski A, Libby P (2011). Interleukin-1β inhibition and the prevention of recurrent cardiovascular events: rationale and design of the Canakinumab Anti-inflammatory Thrombosis Outcomes Study (CANTOS). Am Heart J.

[225] Ridker PM, Everett BM, Thuren T (2017). Antiinflammatory Therapy with Canakinumab for Atherosclerotic Disease. N Engl J Med.

[226] Ridker PM, MacFadyen JG, Thuren T, Libby P (2020). Residual inflammatory risk associated with interleukin-18 and interleukin-6 after successful interleukin-1β inhibition with canakinumab: further rationale for the development of targeted anti-cytokine therapies for the treatment of atherothrombosis. Eur Heart J.

[227] Zhang L, Yang L (2014). Anti-inflammatory effects of vinpocetine in atherosclerosis and ischemic stroke: a review of the literature. Molecules.

[228] Zhang F, Yan C, Wei C (2018). Vinpocetine Inhibits NF-κB-Dependent Inflammation in Acute Ischemic Stroke Patients. Transl Stroke Res.

[229] Bai P, Zhu R, Wang P (2022). The efficacy and safety of fingolimod plus standardized treatment versus standardized treatment alone for acute ischemic stroke: A systematic review and meta-analysis. Pharmacol Res Perspect.

[230] Stoll G, Nieswandt B (2019). Thrombo-inflammation in acute ischaemic stroke - implications for treatment. Nat Rev Neurol.

[231] Nogales C, Mamdouh ZM, List M (2022). Network pharmacology: curing causal mechanisms instead of treating symptoms. Trends Pharmacol Sci.

[232] Casas AI, Hassan AA, Larsen SJ (2019). From single drug targets to synergistic network pharmacology in ischemic stroke. Proc Natl Acad Sci U S A.

